# Lipopolysaccharide biosynthesis and traffic in the envelope of the pathogen *Brucella abortus*

**DOI:** 10.1038/s41467-023-36442-y

**Published:** 2023-02-17

**Authors:** Caroline Servais, Victoria Vassen, Audrey Verhaeghe, Nina Küster, Elodie Carlier, Léa Phégnon, Aurélie Mayard, Nicolas Auberger, Stéphane Vincent, Xavier De Bolle

**Affiliations:** 1 Research Unit in Biology of Microorganisms (URBM), Narilis, University of Namur (UNamur), 61 rue de Bruxelles, 5000 Namur, Belgium; 2 Université de Poitiers, IC2MP, UMR CNRS 7285, Equipe “OrgaSynth”, Groupe Glycochimie, 4 rue Michel Brunet, 86073 Poitiers, France; 3 Bio-organic Chemistry Unit (CBO), Narilis, University of Namur (UNamur), 61 rue de Bruxelles, 5000 Namur, Belgium

**Keywords:** Bacterial physiology, Bacteriology, Pathogens

## Abstract

Lipopolysaccharide is essential for most Gram-negative bacteria as it is a main component of the outer membrane. In the pathogen *Brucella abortus*, smooth lipopolysaccharide containing the O-antigen is required for virulence. Being part of the Rhizobiales, *Brucella* spp. display unipolar growth and lipopolysaccharide was shown to be incorporated at the active growth sites, i.e. the new pole and the division site. By localizing proteins involved in the lipopolysaccharide transport across the cell envelope, from the inner to the outer membrane, we show that the lipopolysaccharide incorporation sites are determined by the inner membrane complex of the lipopolysaccharide transport system. Moreover, we identify the main O-antigen ligase of *Brucella* spp. involved in smooth lipopolysaccharide synthesis. Altogether, our data highlight a layer of spatiotemporal organization of the lipopolysaccharide biosynthesis pathway and identify an original class of bifunctional O-antigen ligases.

## Abstract (web summary)

Pathogenic *Brucella abortus*, containing a mix of lipopolysaccharides with or without O-antigen, grows its envelope in a unipolar manner. Here, Servais *et al*, localize the LPS translocation machinery and identify the main O-antigen ligase in *Brucella* species, shedding light on the basic biology of this organism.

## Introduction

The Gram-negative cell envelope is composed of an inner membrane (IM), a periplasmic space hosting peptidoglycan (PG), and an outer membrane (OM)^1^. The OM constitutes the interface with the external environment. It is an asymmetric bilayer mainly composed of phospholipids in the inner leaflet and lipopolysaccharide (LPS) in the outer leaflet^2,3^. LPS is essential for OM integrity in most Gram-negative bacteria^4^. This amphiphilic molecule is composed of lipid A, a hydrophobic anchor made of acyl chains, a core composed of sugars, and a long polysaccharidic chain, called the O-antigen. Depending on the presence or the absence of the O-antigen, the LPS is referred to as smooth (S-LPS) or rough (R-LPS), respectively. Most rod-shaped bacteria grow and elongate their cell wall in a dispersed manner as the Gram-negative model organism *Escherichia coli*^5,6^. In contrast, bacteria from the Rhizobiales order belonging to the class of alpha-proteobacteria, such as *Brucella abortus*, *Agrobacterium tumefaciens*, and *Sinorhizobium meliloti*, grow unipolarly^7^. New envelope material is incorporated at one pole of the cell (namely the new pole), resulting in a daughter cell exclusively composed of new envelope material^7^. Several proteins were shown to polarly localize at the new or old pole, in agreement with the association of distinct functions at the poles. Among others, Rhizobiales growth and septation (Rgs) proteins were recently localized in *S. meliloti*. For instance, RgsE was shown to be localized exclusively at the new pole. Together with other Rgs proteins, RgsE is thought to be important for polar growth^8,9^. Interestingly, RgsE homolog in *Agrobacterium tumefaciens* was shown to form a polar ring at the new pole and was therefore called Growth Pole Ring protein (GPR)^10^.

Brucellosis is a worldwide zoonosis mainly caused by *B. abortus*, *Brucella melitensis* and *Brucella suis*. These three species are class III pathogens, very close at the phylogenetic level but differing by their host specificity^11^. However, they display S-LPS at the surface which is crucial for their virulent phenotype. In *B. abortus*, PG, two OM proteins (OMP) and LPS were shown to be incorporated at the growth sites, i.e. the new pole and the division site^12^. Moreover, *B. abortus* also displays a mixture of R-LPS and S-LPS on the surface of the smooth wild type^12^. Interestingly, *Brucella* LPS is different from the classical enterobacterial LPS^13^, as for instance, *Brucella* lipid A has one longer acyl chain and its core has a branched structure^13,14^. During LPS biosynthesis, lipid A and the core are assembled at the inner leaflet of the IM to form the R-LPS that is subsequently flipped to the outer leaflet of the IM by the essential ABC transporter MsbA^15^. The O-antigen, a long unbranched homopolymer of 4,6-dideoxy-4-formamide-α-D-mannopyranosyl (also called *N*-formyl-perosamine) found in most *Brucella* strains^16,17^, is the most variable part of the LPS. Its length highly varies within a *Brucella* population and even on individual bacterial cells^18,19^. During its biosynthesis, the O-antigen is linked to a lipid carrier, a 55-carbon isoprenoid, the undecaprenyl phosphate (Und-P)^20^, at the inner leaflet of the IM and is subsequently flipped by a different ABC transporter complex, Wzm/Wzt^21,22^. At the periplasmic side of the IM, an unknown O-antigen ligase links the O-antigen to a fraction of R-LPS to generate S-LPS. R-LPS and S-LPS are then translocated to the OM via the LPS transport machinery (Lpt). The Lpt complex forms a bridge of seven proteins (LptAB_2_CDEFG), spanning from the IM to the OM^23,24^. It has been mainly studied in *E. coli* and all of the proteins constituting the machinery are conserved and essential in *B. abortus*^25^. Until now, the Lpt proteins have never been localized in unipolarly growing bacteria and the O-antigen ligase remains unknown in *Brucella* spp.

Here, we investigate the localization of proteins involved in LPS biosynthesis in the unipolarly growing Rhizobiale, *B. abortus*. We first localize several components of the Lpt machinery in the IM and the OM, as well as the lipid A-core flippase, MsbA, and the O-antigen flippase, Wzm/Wzt. We also identify WadA as a bifunctional enzyme and the main O-antigen ligase in *Brucella* spp. In addition to the cytoplasmic glycosyltransferase activity grafting the last core sugar necessary for the O-antigen ligation, we found that WadA displays an O-antigen ligase activity, proposed to take place in the periplasmic space. Altogether, we propose a model for LPS biosynthesis that involves LPS trafficking from the old pole in the IM to the new pole in the OM of *B. abortus*.

## Results

### The OM component of the Lpt pathway, LptD, is dispersed on *B. abortus* surface

**Fig. 1:**
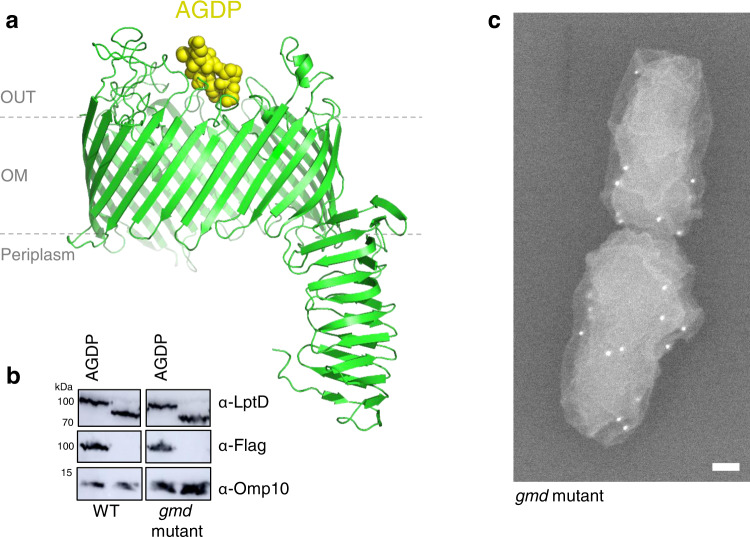
3Flag::LptD is found dispersed on the *B. abortus* cell surface. **a** Three-dimensional model of LptD highlighting the ADGP loop (yellow spheres) in which the 3Flag was inserted. This sequence was among the most variable in the highly conserved LptD sequence (Fig. S1). The 3D structure of LptD was predicted using Swiss model server (https://swissmodel.expasy.org)^62^ and displayed with PyMol v.2.0^63^ **b** The presence of the 3Flag in LptD was assessed by western blot (WB) in the WT and in the *gmd* mutant with polyclonal antibodies directed against LptD and a mAb recognizing the 3Flag. Omp10 was used as a loading control for the blot using α-Flag antibody. All the WB were performed in triplicates with similar results. Source data are provided as a Source Data file. **c** Representative scanning electron microscopy picture for one replicate of *gmd* mutant with *3Flag::lptD* fusion. 3Flag-LptD was detected using a mAb against 3Flag, followed by secondary antibody coupled with gold particles, each white focus corresponds to a single gold particle with the expected size (18 nm) (*n* = 3). Scale bar represents 100 nm. Source data are provided as a Source Data file.

Given that LPS is integrated unipolarly in the OM^12^, we wondered whether the LPS translocation pathway was also localized at the growing cell pole. Together with LptE, LptD forms the main OM part of the Lpt pathway, allowing the last step of the LPS transport to the OM^26,27^. In order to localize this large β-barrel in *B. abortus*, a 3Flag tag was incorporated into a non-conserved loop of LptD (Fig. S1), predicted to be surface exposed (Fig. 1a). The *lptD* gene was replaced by the engineered *3Flag::lptD* fusion, the resulting strain was viable and the fusion protein was detectable (Fig. 1b). Since *lptD* is essential, viability and normal morphology of the engineered strain suggest that 3Flag::LptD is functional. The fusion protein was localized by scanning electron microscopy using an anti-3Flag monoclonal antibody (mAb) and a secondary antibody conjugated to gold particles (Fig. 1c). The fusion could only be detected on the surface of the rough mutant strain (disrupted *gmd*, unable to synthesize the O-antigen)^21^ and not in the wild type (WT) strain, presumably because the long O-antigen impaired the detection of the surface exposed 3Flag tag. The *gmd* mutant with endogenous *lptD* gene was used as negative control to eliminate the gold particles background, i.e. cells with ≤4 gold particles (Fig. S2a, b). The distribution frequency of the gold particles showed that LptD is almost evenly located on the bacterial surface, with a slightly lower frequency near the constriction site (Fig. S2c). In contrast to the polar insertion of LPS, the apparent homogenous LptD localization could be explained by the limited mobility of OMPs as previously shown^12,28^. We therefore hypothesized that LptD could be fed at the growth sites, receiving new LPS molecules by the Lpt proteins located in the IM.

### The IM components of the Lpt complex are localized at the growth sites

**Fig. 2:**
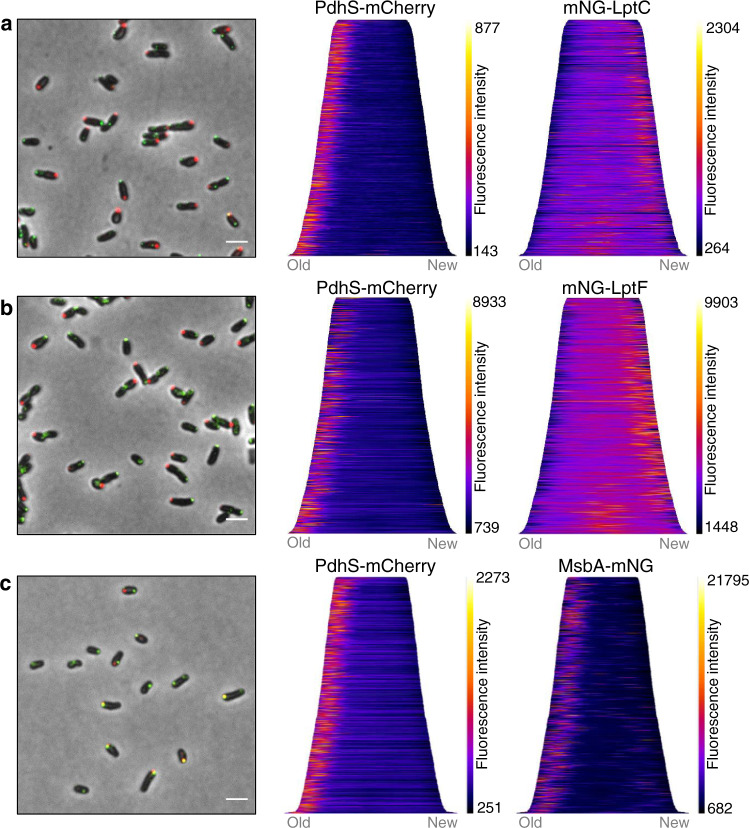
LptC, LptF and MsbA localization. Representative merged microscopy pictures and demograph analyses of (**a**) *mNG-lptC* (*n* = 723 bacteria), (**b**) *mNG-lptF* (*n* = 1398 bacteria), and (**c**) *msbA-mNG* (*n* = 693 bacteria) co-expressing the old pole marker PdhS-mCherry. Both mNG-LptC (**a**) and mNG-LptF (**b**) are detected at the growth sites while MsbA-mNG is localized at the old pole (**c**). Scale bars represent 2 µm. The right panels show demographic representations. The cells were sorted according to their size (from the smallest on top to the largest at the bottom) and oriented according to their cell poles using the old pole marker PdhS-mCherry. Demographs are representative images for one replicate (*n* = 3). Fluorescence intensities are represented as heatmap with minimum and maximum values represented on the vertical-scale, and were automatically selected to provide best signal to background ratio using MicrobeJ image analysis. Source data are provided as a Source Data file for (**a**), (**b**) and (**c**).

The IM components of the Lpt transport machinery include LptB, LptC, LptF and LptG^23^, which all have a distinct and essential homolog in *B. abortus*^25^. The localization of LptC was achieved by fusing mNG at its N-terminus (*mNG-lptC*), i.e. the cytoplasmic part of the protein, by allelic replacement at the chromosomal locus. The *mNG-lptC* strain was viable, grew similarly to the WT in rich medium and still displayed unipolar growth indicating a functional fusion (Fig. S3a, b). The marker PdhS-mCherry was used to determine the old pole^29^. LptC was mainly found at the new pole in non-divisional bacteria, or at the mid cell in pre-divisional cells (Fig. 2a), similar to the LPS incorporation profile. Demographic analysis confirmed this localization pattern (Fig. 2a). Since LptC forms a stable complex with LptB_2_FG in other bacteria^30^, we also investigated the localization of mNG-LptF to confirm that another component of the IM complex of the Lpt system is co-localizing with LPS insertion sites. The strain *mNG-lptF* was constructed by allelic replacement and showed unipolar growth (Fig. S3). The mNG-LptF localization pattern was comparable to mNG-LptC (Fig. 2b), further supporting that the whole IM complex would be found at the new pole and the division site.

**Fig. 3:**
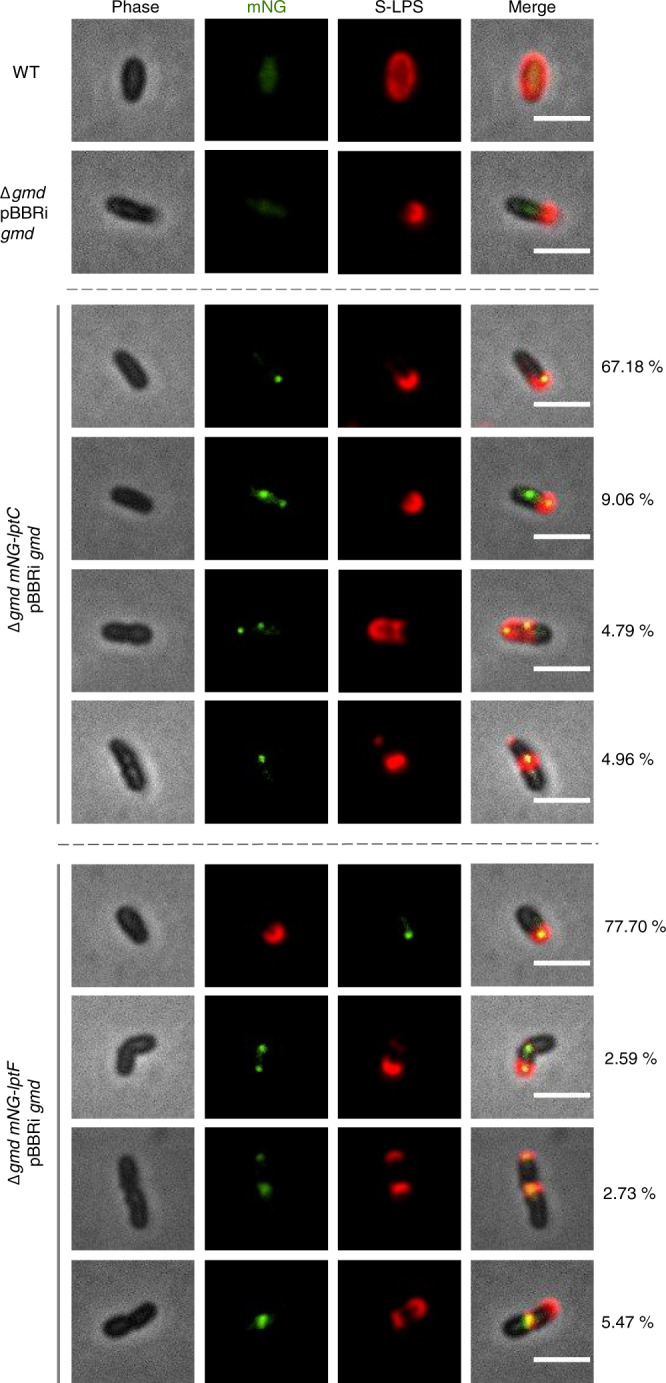
S-LPS induction in *mNG-lptC* and *mNG lptF*. The *gmd* synthesis was induced upon IPTG addition in exponential phase culture and the bacteria were labelled for S-LPS using the monoclonal antibody B66/04F09 targeting the *B. abortus* O-antigen. In total, 94.36 or 94.09% of the bacteria displayed colocalization signal for S-LPS and LptC or LptF respectively. Most of the bacteria were found with one focus of LptC or LptF colocalising with S-LPS incorporation at the same pole or colocalizing at the division site. The WT strain was used as a positive control for O antigen labelling and the Δ*gmd*::pBBRi *gmd* strain was used as a positive control of induction and polar detection of newly inserted S-LPS. *N* = 585 bacteria for *mNG-lptC* and *n* = 688 bacteria for *mNG-lptF*. The analysis represents three biological replicates. Scale bar is 2 µm. Source data are provided as a Source Data file.

We wanted to further assess whether LptC and LptF found at the growth areas are involved in new LPS transport to the OM. To answer that question, we induced the O-antigen synthesis in strains having a *mNG-lptC* or *mNG-lptF* fusion and labelled them for S-LPS synthesis. These strains were deleted for *gmd* (Δ*gmd*) and a copy of *gmd* was placed under the control of *Escherichia coli* P_*lac*_ inducible promoter to regulate O-antigen synthesis, as previously reported^12^. The *gmd* synthesis was induced and the bacteria were labelled for S-LPS incorporation. The WT strain was used as positive control for S-LPS detection and a *gmd* mutant only carrying the plasmidic P_*lac*_-*gmd* fusion was used as positive control of induction. Only the bacteria labelled for S-LPS and displaying a focus for LptC or LptF were taken into account for the analysis. S-LPS signal was co-localizing for at least one focus of mNG-LptC or mNG-LptF in 94.36% and 94.09 % of the bacteria for *mNG-lptC* and *mNG-lptF* respectively (Fig. 3 and Table S7), supporting incorporation of S-LPS at the growth sites in strains having a signal for mNG-LptC or mNG-LptF at the same area.

### LptCF localization is conserved in the Rhizobiale *Agrobacterium tumefaciens*

Rhizobiales have a similar growth pattern^7^, which suggests that they may involve similar localized molecular machineries for the biosynthesis of their envelope. The GPR protein of *A. tumefaciens* and its homolog RgsE in *S. meliloti* both display a localization to the growth pole, suggesting that Rhizobiales may indeed have homologous essential proteins with a similar localization pattern. Here, we localized the essential RgsE homolog^25^ in *B. abortus* by C-terminal fusion with the mNG and the resulting strain (*rgsE-mNG)* was studied in a *pdhS-mCherry* background^29^. Demograph analysis revealed that RgsE was exclusively localized at the new pole (Fig. S4), similarly to *S. meliloti* and *A. tumefaciens*.

**Fig. 4:**
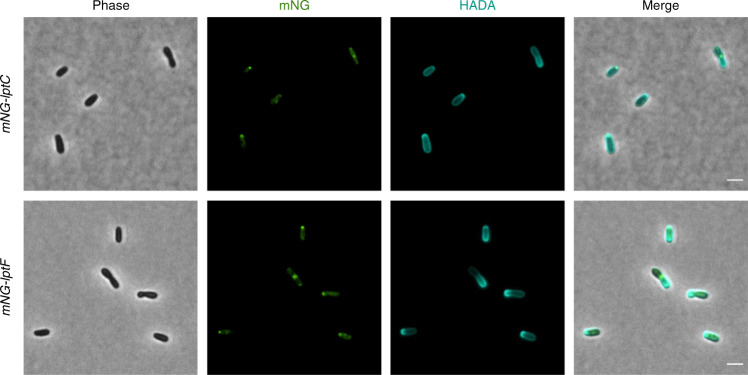
LptC and LptF localization in *A. tumefaciens*. Both mNG-LptC and mNG-LptF are localized at the new pole and the constriction site in *A. tumefaciens*. Bacteria were labelled with HADA to highlight the new pole in Rhizobiales. The experiment was performed three times for each strain, giving similar results. Scale bar represents 2 µm. Source data are provided as a Source Data file.

We next wondered if the localization of Lpt IM proteins that we observed in *B. abortus* was conserved in *A. tumefaciens*. The localization of both LptC and LptF was obtained by fusion with the mNG at the cytoplasmic N-terminal part of the protein and the fusion was performed at the chromosomal locus in *A. tumefaciens*. To distinguish the poles, we took advantage of the HADA labelling which highlights new peptidoglycan insertion sites, i.e. the new pole for *A. tumefaciens*^31^. LptC and LptF were mainly found at the new pole and the constriction site (Fig. 4), just as in *B. abortus*, suggesting that the IM Lpt complex localization would be conserved in the Rhizobiale order, together with other conserved essential proteins, like RgsE/GPR, that are also homologous among the Rhizobiales.

### The essential flippase MsbA is mainly found at the old pole

Since the IM Lpt proteins are localized at the growing zones, it was tempting to hypothesize that the molecular actors involved in the LPS biosynthesis upstream of the Lpt, such as MsbA, would be in a close vicinity to facilitate LPS translocation through the Lpt system. Therefore, a *msbA-mNG* fusion was constructed and integrated at the chromosomal locus to replace the endogenous essential *msbA* gene. This genetically modified strain was viable, displayed a normal growth in rich medium as well as unipolar growth (Fig. S3), suggesting that MsbA-mNG is functional. MsbA-mNG localization was analyzed in a *pdhS-mCherry* background. Surprisingly, MsbA was found at the old pole in 97% of the bacteria (Figs. 2c and S5), which was in total contradiction with our expectations. Manual counting highlighted that 52% of the bacteria only presented one focus at the old pole only, while 47% of them presented at least one additional clearly distinct focus throughout the whole bacterium in addition to the old pole (Fig. S5).

**Fig. 5:**
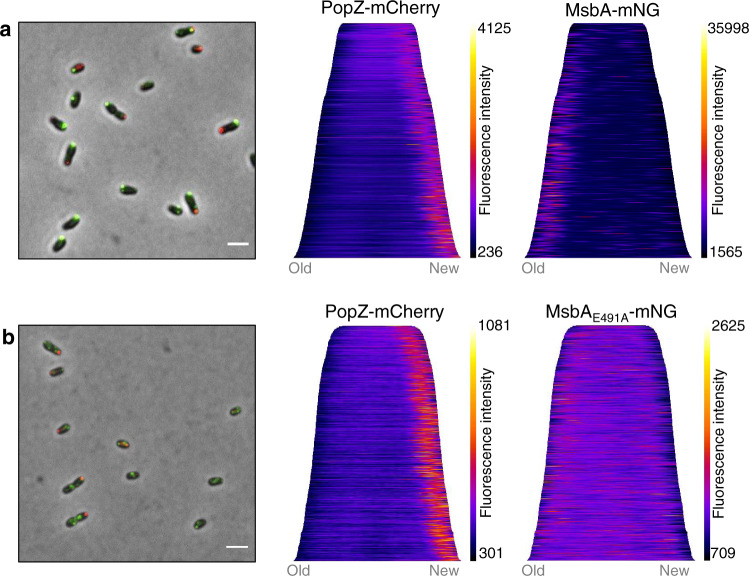
Mutation in the Walker B motif of MsbA leads to diffused localization. Representative merged pictures and demograph analyses for two strains (*n* = 3). **a** An additional copy of MsbA fused to mNG (pSK_*msbA-mNG*) was still mainly localized at the old pole. The two demographs represent PopZ-mCherry (new pole marker) and MsbA-mNG localization (*n* = 1095 bacteria). Source data are provided as a Source Data file. **b** An additional copy of MsbA mutated (E491A) and fused to the mNG. MsbA_E491A_-mNG (pSK_*msbA*_*E491A*_*-mNG*) showed in general a less intense and more dispersed signal. Demograph analyses represent PopZ-mCherry and MsbA_E491A_-mNG localization respectively (*n* = 536 bacteria). Fluorescence intensities are represented as heatmaps with automatically selected minimum and maximum values represented on the scale in order to provide the best signal-to-background ratio by MicrobeJ. Scale bars are 2µm. Source data are provided as a Source Data file.

In a next step, we were wondering if MsbA activity was coupled to its old pole localization. To address this question, we generated a strain in which *msbA* was duplicated. The strain contains an intact copy of *msbA* together with a *msbA-mNG* fusion (pSK_*msbA-mNG*) and the PopZ-mCherry new pole marker^32^. This strain displayed normal growth and MsbA localization pattern comparable to the strain with a single *msbA-mNG* gene (Fig. 5a). Furthermore, we constructed a strain in which the additional *msbA-mNG* gene was replaced with a *msbA-mNG* allele in which the glutamate 491 codon was replaced by an alanine (pSK_*msbA*_E491A_-*mNG*). As already described for other ABC transporters, mutating the corresponding glutamate on the Walker B Motif impairs ATP hydrolysis, thereby blocking the protein in an inactive ATP-bound conformation^33^. Interestingly, MsbA_E491A_-mNG localization was dispersed and remaining foci were much less intense (Fig. 5b), suggesting that the proper localization of MsbA is coupled to its ATPase activity.

### WadA is the O-antigen ligase in *Brucella* species

In an effort to localize other proteins involved in S-LPS synthesis, we realized that although the whole LPS translocation pathway has been identified, one important biosynthesis enzyme was still missing. Indeed, the O-antigen ligase, attaching the O-antigen to the lipid A-core using Und-PP-O-antigen as a substrate, remains unknown in *Brucella* spp. We identified two putative candidates, WaaL and WadA, that were predicted to code for O-antigen ligases based on domain composition. WaaL has been identified as the main O-antigen ligase in many bacterial species^34–37^. However, deletion of the *waaL* homolog in *B. abortus* resulted in smooth phenotype like the wild type (Fig. S6), suggesting that WaaL is not the main O-antigen ligase under the tested conditions.

**Fig. 6:**
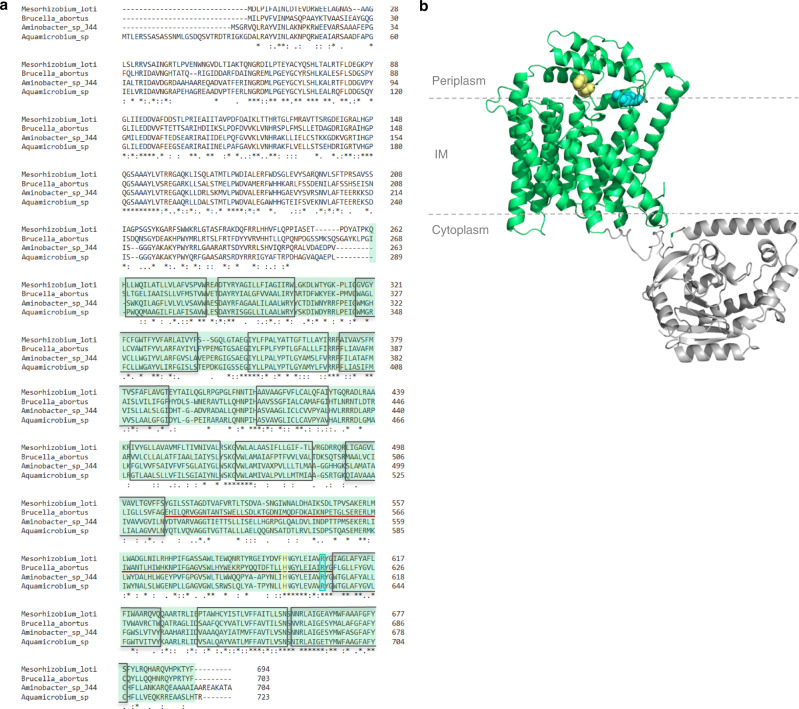
Sequence alignment and structure prediction for WadA as the O-antigen ligase in *B. abortus*. **a** Sequence alignment of WadA of *B. abortus*, and WadA homologs of Rhizobiales. Homologs were found using Delta-Blast^63^ and the sequence alignment was performed using Clustal Omega (1.2.4)^64,65^. An asterisk (*) indicates a fully conserved amino acid, a colon (:) indicates amino acids with strongly similar properties, and a period (.) indicates amino acids with weakly yet similar properties at the position. The boxes represent transmembrane helixes predicted by DeepTMHMM (v1.0.8)^66^ and the red line is the main periplasmic loop of WadA identified in *B.* *abortus*. Arg-614 (R614, cyan) was mutated in this study. Highly conserved His (H605, yellow) was shown to mediate O-antigen ligase activity with a distant homolog^41^. From amino acid 1 to 266 is the glycosyl transferase domain and in green from the amino acid 267 to 703 of *B. abortus* is the predicted O-antigen ligase domain. **b** Three dimensional model generated by Alpha-fold^38, 39^, displayed with PyMol^67^ and modified to avoid the GT domain to be positioned in the IM. WadA comprises both the glycosyl-transferase (in grey) and the O-antigen ligase domain (in green) which presents 12 transmembrane (TM) segments (boxes in the alignment) and 3 periplasmic loops. In cyan, R614 is found in the main periplasmic loop (red line in the alignment), facing the H605 also found in the periplasmic loop.

**Fig. 7:**
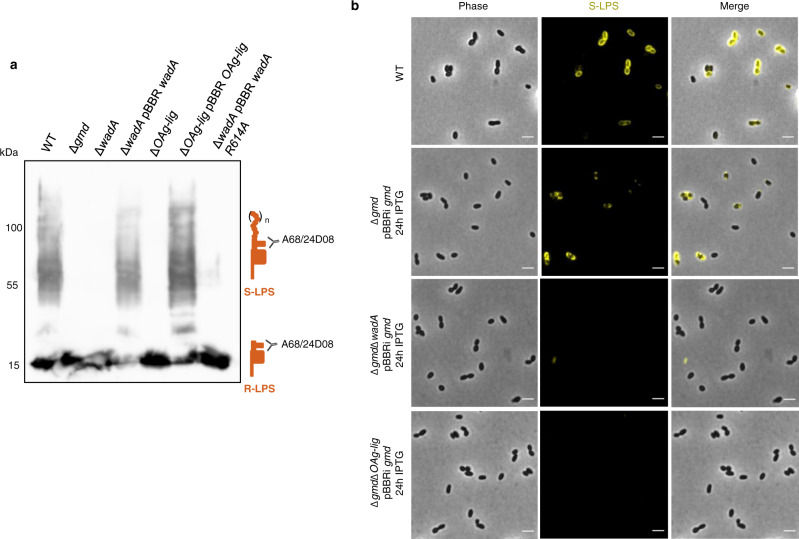
WadA is the main O-antigen ligase in *Brucella* spp. **a** WB analysis using an antibody against the core of the LPS (A68/24D08) in *B.* *melitensis* 16M mutants. WB was performed in biological triplicates giving similar results (*n* = 3). Source data are provided as a Source Data file. **b** Immunofluorescence microscopy labelling the S-LPS (A76/12G12) of the WT *B.* *abortus* 544, Δ*gmd*Δ*wadA*, and the Δ*gmd*Δ*OAg-lig* expressing *gmd* upon IPTG induction for 24 h. Picture is shown for one representative experiment (*n* = 3). Scale bars are 2 µm. Source data are provided as a Source Data file.

Three dimensional structure prediction of *Brucella* WadA using Alpha-Fold^38,39^ highlighted 12 transmembrane domains with periplasmic loops (Fig. 6), similar to the *E. coli* O-antigen ligase topology^40,41^. The *wadA* gene was scored as essential in *B. abortus* 2308^25^, while its ortholog was not essential in *B. melitensis*^42^. However, these two *wadA* sequences are identical on a sequence level. Although very close phylogenetically, *B. abortus* and *B. melitensis* display differences regarding their OM. For example, *B. abortus* presents a large deletion of 17 kb in genes presumably involved in LPS biosynthesis and regulation^43^. Moreover, major OMP such as Omp31 are absent in *B. abortus* but not in *B. melitensis*^44^ and their O-antigen is different^11^, suggesting that the OM composition and the regulation of envelope biosynthesis might be different in these two species. In *B. melitensis*, WadA acts as the glycosyltransferase that adds the terminal core sugar (a glucose) needed as attachment structure for the O-antigen^45^. The rough phenotype of *B. melitensis wadA* deletion mutant (Δ*wadA*) was confirmed both by western blot (WB) analysis (Fig. 7a) and immunofluorescence microscopy (Fig. S7). WadA contains two domains, a cytoplasmic N-terminal glycosyltransferase (GT) domain and a predicted C-terminal O-antigen ligase (OAg-lig) domain (Fig. 6). Deletion of a major part of the largest periplasmic loop of the *OAg-lig* domain (Δ*OAg-lig*) was constructed (see Material and Method and Fig. 6a). The Δ*OAg-lig* mutant displayed a rough phenotype detected by WB analysis using the core targeting antibody (A68/24D08), allowing the distinction between R-LPS and S-LPS after electrophoresis (Fig. 7a). The Δ*OAg-lig* mutant could be complemented by adding a copy of the *OAg-lig* domain coding sequence (Fig. 7a), suggesting that the OAg-lig domain is indeed coding for a functional O-antigen ligase in *B. melitensis*.

To further support the role of WadA as the main O-antigen ligase in *Brucella*, we mutated an arginine (Arg-614) conserved in several WadA homologs among the Rhizobiales (Fig. 6). Arg 614 is located at the terminal region of the largest periplasmic loop of the OAg-lig domain (Fig. 6). Indeed, analysis of *E. coli* O-antigen ligase revealed a cluster of positively charged residues in the periplasmic loop that were required for O-antigen ligation^40,46^. Complementation of Δ*wadA* with *wadA* R614A lead to very low levels of S-LPS synthesis, while complementation with intact *wadA* restores the WT S-LPS phenotype (Figs. 7a and S7). A recent study showed that several other residues were involved in O-antigen ligation^41^, which could explain why some residual S-LPS can be detected in the presence of the WadA-R614A mutant.

It was previously shown that alteration of the O-antigen synthesis leads to growth defects in *E. coli*^47^. Since the Und-P lipidic carrier binds both O-antigen and PG precursors, alteration of the free Und-P pool could also impair PG synthesis and among others bacterial growth^47^. We therefore hypothesized that the essentiality of *wadA* in *B. abortus* could be due to an indirect effect of Und-P sequestration by the O-antigen. Indeed, *ΔwadA* and *ΔOAg-lig* deletions strains could be obtained in a Δ*gmd* strain unable to synthesize the O-antigen. In order to show that WadA and more particularly its OAg-lig domain is required for the production of S-LPS in *B. abortus*, we introduced the *gmd* gene in the Δ*gmd*Δ*wadA* and Δ*gmd*Δ*OAg-lig* strains, to test the hypothesis that ∆*wadA* and ∆*OAg-lig* mutations would impair S-LPS synthesis even when *gmd* is expressed. A copy of *gmd* was added under the control of the p_*lac*_ promoter in the ∆*gmd*, Δ*gmd*Δ*wadA* and Δ*gmd*Δ*OAg-lig* strains. As expected, immunofluorescence microscopy after induction of *gmd* expression showed no fluorescence signal for S-LPS neither in Δ*gmd*Δ*wadA* nor in Δ*gmd*Δ*OAg-lig* whereas S-LPS labelling could be detected in Δ*gmd* after induction used as positive control (Fig. 7b).

### WadA and Wzm are dispersed in the IM

**Fig. 8:**
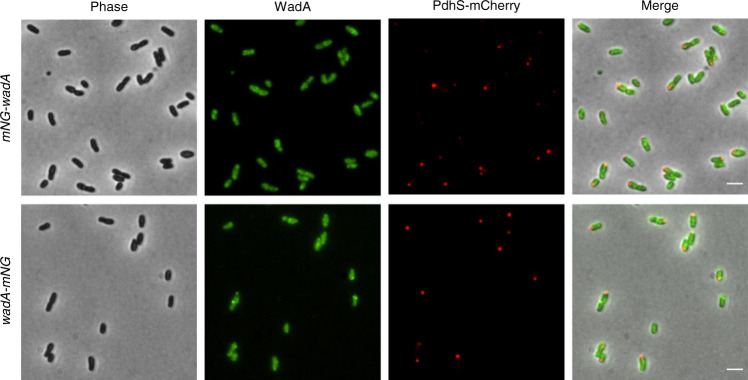
WadA localization. Representative picture for *mNG-wadA* and *wadA-mNG* in a strain co-expressing Pdhs-mCherry, observed in exponential phase of growth. Both strains were observed in biological triplicates. The scale bar represents 2 µm. Source data are provided as a Source Data file.

In light of the previous localization of MsbA and the Lpt pathway, we would expect WadA to be localized all over the IM to allow the grafting of the O-antigen along the trafficking from the old pole to the growth zone (new pole or division site). Actually, WadA would first have to exert its GTase activity at the cytoplasmic leaflet, before the Lipid A-core and the O-antigen are flipped by MsbA and Wzm/Wzt respectively, and second its O-antigen ligase activity at the periplasmic leaflet. In order to localize WadA, we fused *wadA* to the *mNG* at the chromosomal locus. The fusion was constructed at the N-terminal (*mNG-wadA*) and at the C-terminal part (*wadA-mNG*) of the protein (see Methods for more details). The strains were not affected for growth and still displayed unipolar growth meaning that the fusions are functional. For both mNG-WadA and WadA-mNG, the signal always appeared as weak foci rather dispersed, sometimes surrounding the bacterial cell. No precise pattern of localization could be observed for WadA (Fig. 8). Therefore, we hypothesized that WadA would be dispersed on the IM.

Interestingly, Wzm, one of the two subunits of the ABC transporter of Und-PP-O-antigen, showed a similar pattern to WadA, with a heterogeneous distribution surrounding the bacteria, suggesting a dispersed localization of the transporter at the IM (Fig. S8), very similar to the WadA fusions to mNG. The *mNG-wzm* fusion was constructed at the chromosomal locus and the strain grew and displayed a morphology similar to the WT strain.

## Discussion

**Fig. 9:**
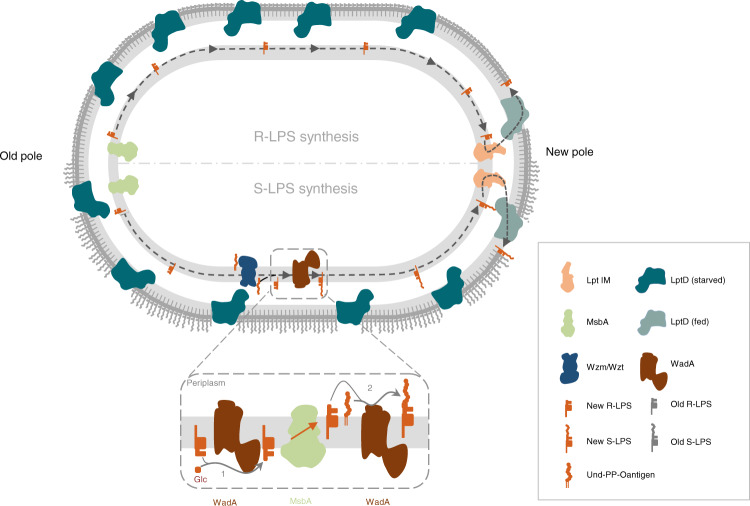
Schematic model localizing different actors involved in LPS synthesis and translocation of *B. abortus*. When synthesized, the R-LPS is anchored to the inner leaflet of the IM. Then, the essential ABC transporter MsbA mainly localized at the old pole flips the R-LPS to the outer leaflet of the IM. The R-LPS is able to diffuse within the IM to the active growth sites (here the new pole) where the LPS translocation pathway, consisting of LptC and LptF is mainly localized. The R-LPS is translocated from the outer leaflet of the IM to the outer leaflet of the OM by the IM Lpt complex, LptA and finally the LptDE translocon. In this model, LptD localized at the growth site (light blue LptD) would incorporate new LPS molecules into the outer leaflet of the OM, whereas homogenously dispersed LptD previously incorporated at the OM would remain starved for LPS (dark blue LptD). On the lower part of the model, lipid A-core is synthesized and anchored in the inner leaflet of the IM and the GTase domain of WadA adds the last core sugar (Glc, Glucose) (1), thereby forming the R-LPS. The R-LPS is then flipped by MsbA and at the periplasmic leaflet the O-antigen is ligated by WadA periplasmic O-antigen ligase domain to form S-LPS (2), which would diffuse in the IM until it reaches the Lpt system in order to be exported to the OM. WadA and Wzm are distributed all along the IM.

During growth, Gram-negative bacteria must expand the different layers of their cell envelope, PG and OM, in a temporal and spatial coordinated manner. Similar to other bacteria of the Rhizobiales order, *B. abortus* displays unipolar growth by incorporating new envelope components at the new pole in non-divisional bacteria and at the constriction site in divisional bacteria. Previously, LPS was one of the components reported to be unipolarly incorporated at the OM of *B. abortus*^12^. However, to the best of our knowledge, the essential LPS translocation pathway has never been localized before. Here, we show that although the main OM component of the Lpt pathway, LptD, is found dispersed at the bacterial surface, the IM components LptC and LptF are localized at the growth sites, in accordance with the LPS incorporation sites (Fig. 9). LptC and LptF polar localization is conserved in *A. tumefaciens*, suggesting that LPS incorporation at the new pole may be a shared feature among Rhizobiales, just as the presence of essential proteins proposed to be involved in polar growth, such as RgsE/GPR. How to explain that strikingly, LptD localization does not align with the LPS insertion sites in *B. abortus*? When a component like LptD is inserted in the OM, it would remain fixed at its incorporation site. A static OM was previously suggested by the analysis of Omp2b porin, Omp25 and LPS^12^, and by the covalent attachment of several OMPs to PG^48^. Recent data by Vollmer and colleagues highlighted that although dispersed at the OM, the Bam complex would only be active at growth areas in *E. coli*^49^. Bam activity and OMP assembly is regulated by the maturation state of the PG, as the binding of old tetrapeptide inhibits the foldase activity of the machinery, leading to spatiotemporal coordination of PG and OM growth^49^. Similarly, LptD would only incorporate LPS during growth when it is fed with LPS substrates from the IM Lpt proteins, LptC and LptF, shown to be localized at the growth sites. By allowing the assembly of the whole Lpt complex, the IM proteins would determine the LPS incorporation sites and would further contribute to the growth coordination of the three layers of the bacterial cell envelope because new PG and new OMP β-barrels are thought to be incorporated at the same sites^12^. How LptC and LptF are targeted to the growth sites and thereby feed LptD still remains to be discovered. Furthermore, it is not excluded that LptD located outside the canonical growth areas could occasionally be fed by the IM proteins or the periplasmic LptA, for example in strains that produce blebs and thus need to compensate for the loss of OM fragments everywhere on their surface^48^. In that regard, having newly synthesized LPS available for export at any place in the OM would be advantageous.

Since MsbA is mainly localized at the old pole and LptD is dispersed in the OM, we propose that the entire LPS transport system is not restrained at the growth areas in *B. abortus*. The analysis of a non-functional variant of MsbA suggests that MsbA function is coupled to its localization. As a fraction of MsbA-mNG signal was found outside the old pole, it could be possible that this protein has a degree of movement depending on its activity, such as the ability to catch a substrate at the old pole and deliver it outside the old pole. Another attractive hypothesis would be that the old pole localization of MsbA is driven by the availability of its substrates. In both cases, the old pole localization of MsbA could be explained by the localization of the proteins involved in lipid A-core synthesis, but this needs to be further investigated. A main flippase activity at the old pole implies that lipid A-core needs to be mobile in the outer leaflet of the IM, from the old pole to the new pole of the cell, in non-dividing bacteria (Fig. 9). This hypothesis is consistent with previous observations in *E. coli* suggesting that the whole IM is relatively fluid since it could be mixed in about 50 s^50^. These data reveal an unexpected aspect of LPS biosynthesis, involving trajectories in the IM and specific localization of enzymes, flippases and transporters that would be interesting to investigate in other bacteria.

Previous WadA characterization in *B. melitensis* convincingly showed that it is responsible for the addition of a glucose as the terminal sugar of the core, i.e. the anchor for the O-antigen^42^. Here we show that the C-terminal domain of WadA, which is predicted to be an integral IM part of the protein with several periplasmic loops (Fig. 6), functions also as an O-antigen ligase in *B. melitensis* and *B. abortus*. To the best of our knowledge, it is the first time that a bifunctional O-antigen ligase is identified and also that a bifunctional membrane spanning enzyme, with catalytic sites on both sides of the membrane, is described. WadA is proposed to add the terminal sugar on the core of LPS at the cytosolic side of the IM, and subsequently ligate the O-antigen once LPS precursor has been flipped over the IM by MsbA. Such a dual activity would ensure the grafting of an appropriate sugar (i.e. glucose) on a conserved part of the core (3-deoxy-D-manno-oct-2-ulosonic acid, KDO) to subsequently attach the O-antigen on this glucose, and thus facilitate the functionality of the O-antigen ligase after an event of horizontal gene transfer. Indeed, it was already suggested that O-antigen ligases display a so-called “relaxed specificity” towards O-antigen and would rather be specific to the core structure of the lipid A-core than to the O-antigen^51,52^. In that regard, displaying both GTase and O-antigen activity on one protein would increase the probability for a successful gene transfer. Indeed, the GTase activity in the cytoplasm, acting on a very conserved KDO residue, prepares the substrate for the O-antigen ligase activity in the periplasm. Interestingly, many genes involved in the O-antigen synthesis in *Brucella* were already suggested to be acquired by horizontal gene transfer, as their GC content is much lower (about 48%) as compared to the rest of the genome (58%), and *wadA* displays a GC content of 51%^21,53^. Although few WadA homologs were identified in available databases, this bifunctional action mode could be found in other O-antigen ligases as well. WadA homologs display conserved residues, including some positively charged residues in the predicted periplasmic loop, and our data suggest that Arg614 plays an important role in the O-antigen ligase activity. WadA is thus forming a new class of O-antigen ligases, probably distantly related to previously described O-antigen ligases since WadA could be identified with a low similarity to existing O-antigen ligases. Finally, WadA being dispersed at the IM could mean that the lipid A-core would be able to interact with the enzyme anywhere at the IM before and after flipping by MsbA. Since Wzm is also found dispersed in the IM, one could imagine that O-antigens are available everywhere at the IM, ready to be ligated onto the last core sugar of the lipid A-core. The export of LPS to the OM excludes the substrate from WadA, thus having MsbA at the old pole could have been selected to increase the probability of ligation of the O-antigen on R-LPS, thus increasing the proportion of S-LPS on the surface of the bacterium. This would make sense since the O-antigen was shown to be crucial for the success in various infection models in *Brucella* species^54–57^.

In conclusion, this work highlights a new layer of spatiotemporal regulation of proteins involved in LPS synthesis and transport. Moreover, it reveals a new class of bifunctional O-antigen ligases. The investigation of homologous systems in other Gram-negative bacteria could be of great interest.

## Methods

### Bacterial strains and media

*Brucella abortus* 544 WT resistant to Nalidixic acid (Nal^R^) and derivative strains were grown in TSB rich medium (3% Bacto Tryptic Soy Broth) at 37 °C. *Brucella melitensis* (Nal^R^) WT and derivative strains were grown in 2YT rich medium (1% yeast extract, 1.6% peptone, 0.5% NaCl) at 37 °C. *Agrobacterium tumefaciens* C58 WT and derivative strains were grown in LB rich medium at 30 °C. *Escherichia coli* DH10B and S17 strains were cultivated in LB medium at 37 °C. All the strains used in this study are listed in Table S1.

When necessary antibiotics were added at the following concentrations; kanamycin (Kan, 10 or 50 µg/ml, at the chromosomal locus or on a plasmid respectively), chloramphenicol (Cm, 4 or 20 µg/ml), nalidixic acid (Nal, 25 µg/ml) and ampicillin (Amp, 100 µg/ml).

### Strain construction

For deletion strains, about 500 base pairs upstream and downstream the region to be deleted were amplified and assembled by overlapping PCR. For Δ*OA-lig*, a major part of the main periplasmic loop was deleted, from amino acid 534 to 612. Translational fusions with the mNG were performed on the N-terminal or C-terminal part of the protein depending on the construct. Important to note, the mNG sequence was adapted on the codon bias of *Brucella*. Briefly, about 500 base pairs upstream and downstream the region of interest and the mNG gene were amplified by PCR. Except for RgsE and *Agrobacterium strains* that were constructed using the Gibson assembly (see below), all the constructs were made by overlapping PCR, restriction and ligation in the corresponding plasmid. Apart from for RgsE, a 3Flag tag was inserted downstream the mNG start codon. For LptC, the poorly conserved N-terminal part of the protein, TADIPHIHVP, was duplicated and used as a linker. For all the other constructs, RSATGS was used as a linker between the gene of interest and the mNG. Fusion and deletion strains were constructed by allelic replacement on the chromosomal locus as previously described using a pNPTS138 integrative plasmid (Kan^R^)^32^. For *Agrobacterium* selection, Ampicillin was used at the knock in step –instead of nalidixic acid. The additional copies of MsbA-mNG and MsbA_E491A_-mNG were provided on a pSK plasmid (Kan^R^), and was integrated in the genome via homologous recombination. Complementation strains were constructed using a pBBR1MCS replicative plasmid carrying a chloramphenicol resistance cassette and the genes of interest were under the control of their endogenous promoter. In particular, for the complementation of the *OAg-lig* domain, overlapping PCR fusing the promoter of *wadA* to the internal region of *wadA* coding for the OAg-lig domain (aa from 267 to 703) were made so that the OAg lig was under the control of *wadA* endogenous promoter. For the induction of *gmd* expression a pBBR containing *gmd* under the control of the P_*lac*_ promoter was used, as previously described^12^. If required, the old pole marker or the new pole marker were integrated on a pSK (Kan^R^) or pKS(Cm^R^) respectively. All the primers and plasmids and ORFs used in this study are listed in Tables S2, S3 and S6 respectively.

### Gibson assembly

Plasmid and inserts were amplified by PCR using primers with 20 overlapping base pairs designed with Benchling. The Gibson reaction was assembled on ice with 10 µl of the 2× Gibson mix to which the plasmid (100 ng) and inserts were added in a 1:1:1 ratio. Then, the reaction was kept at RT for 30 s and incubated overnight at 50 °C. The 2X Gibson mix was made *in house*. For 800 µl final volume, 0.6 µl T5 exonuclease (10 U/µl, NEB), 20 µl Phusion DNA polymerase (2 U/µl, NEB), 160 µl *Taq* DNA ligase (40 U/µl, NEB), 320 µl 5x reaction buffer (see below) and 300 µl of water. The 5× reaction buffer consisted of 0.5 M Tris-HCl, 50 mM MgCl_2_, 1 mM of each dNTPs, 50 mM DTT, ¼ W/V PEG 8000, 5 mM NAD and dH_2_O.

### Induction of S-LPS

For the inducible strains of *B. abortus* 544 Δ*gmd* P_*lac*_-*gmd*, Δ*gmd*Δ*wadA* P_*lac*_-*gmd* and Δ*gmd*Δ*OAg-lig* P_*lac*_-*gmd*, early exponential phase culture (OD_600_ 0.2) were split in two parts; in TSB supplemented with 1 mM of IPTG (induced) and in TSB without IPTG (non-induced). The cultures were incubated for 24 h at 37 °C under shaking. Then, bacteria were labelled with mAb against S-LPS (A76/12G12, see below) and analyzed by fluorescence microscopy. The same protocol was used for Δ*gmd mNG-lptC* P_*lac*_-*gmd* and *mNG-lptF* P_*lac*_-*gmd*, except that the cultures were induced for 9 h with 1 mM of IPTG and labeled for S-LPS with B66/04F09 antibody.

### IF

The S-LPS was visualized by IF using specific mAb against the O-antigen (undiluted A76/12G12 or B66/04F09 depending on the experiment). Exponential phase cultures were washed 2 times in PBS at 8200 × *g*. for 2.5 min, and resuspended in the supernatant for the hybridoma culture containing the mAb. After 40 min of incubation at RT on a rotating wheel, the samples were washed 3 times in PBS and the pellet was resuspended in the secondary anti mouse antibody coupled to AlexaFluor 514 (1:500, in PBS), and incubated at RT on the rotating wheel for 1 h. Bacteria were then washed 3 times, resuspended in PBS and analyzed by fluorescence microscopy.

### eFluor labelling

Bacteria in early exponential phase (OD_600_ 0.2) were washed twice in phosphate buffered saline (PBS) and resuspended in eBioscience™ Cell Proliferation Dye eFluor™ 670 (eFluor, Invitrogen) at a final concentration of 1 μg/ml in PBS. After 15 min of incubation protected from light, bacteria were washed 3 times, resuspended in preheated TSB medium. After 3 h of incubation at 37 °C under constant agitation, the bacteria were washed 3 times and resuspend in PBS and analyzed by fluorescence microscopy.

### HADA labelling

Fifty µl of *A. tumefaciens* bacterial culture in exponential phase were labelled with HADA (final concentration 500 µM) for 10 min at 30 °C with shaking and protected from light. The bacteria were washed two times in PBS for 3 min at 8200 × *g*. The pellet was then resuspended in 50 µl of 70% EtOH at 4 °C incubated on ice for 10 min for fixing the cells. Bacteria were washed four times in PBS, resuspended in PBS and observed by fluorescence microscopy.

### Microscopy and analysis

Exponential phase bacterial cultures were washed two times and resuspended in PBS. Two µl of the bacterial suspension were spotted onto 1% agarose pads. Images were acquired using a Nikon Eclipse Ti2 equipped with a phase-contrast objective Plan Apo λ DM100XK 1.45/0.13 PH3 and a Hamamatsu C13440-20CU ORCA-FLASH 4.0. Fluorescence images were analyzed using FIJI v.2.0.0^58^, a distribution of ImageJ. Look-up tables (LUT) were adjusted to the best signal–background ratio. For demograph analysis, bacteria were detected and analyzed using MicrobeJ, a plugin of ImageJ software^59^. Only isolated bacteria were taken into account, aggregates were excluded from the analysis. If necessary, small aggregates or incorrectly selected bacteria were manually removed from the analysis. Whether the strains contained the old (PdhS-mCherry) or new pole marker (PopZ-mCherry), only the bacteria containing one focus were retained for the analysis. Due to technical variability (e.g. thickness of the agarose pad), different time of exposure, LUT, tolerance for focus detection and minimal fluorescence intensity were used for the different analysis. Replicates of microscopy pictures in Figs. 1c, 2–5, 7b, 8; S3b, S4, S6b, S7 and S8 are available on Figshare (10.6084/m9.figshare.c.6383685.v1).

### Western blot analysis

Exponential phase cultures were normalised to OD_600_ 10 in PBS and inactivated for 1 h at 80 °C. SDS-β-mercaptoethanol loading buffer was added at 1:4 of final volume. Samples were heated at 95 °C for 15 min and were loaded on 12% acrylamide gels. After migration, proteins and LPS were transferred onto a nitrocellulose membrane (GE Healthcare Amersham Protran 0.45 NC) and blocked overnight at 4 °C in PBS supplemented with 0.05% Tween-20 (VWR) and 5% (w/v) milk (Nestlé, Foam topping). Membranes were washed three times for 10 min in PBS 0.05% Tween-20. Afterwards, membranes were incubated for 1 h at room temperature (RT) with primary antibodies (anti-core-LPS A68/24D08/G09 and anti-3Flag FG4R (Thermo Fisher Scientific), 1:1000; anti-LptD, 1:5000) (Table S4). Membranes were then incubated for 1 h at RT with the corresponding secondary horseradish-peroxidase-coupled antibody (1:5000, Table S5). Both antibodies were diluted in PBS supplemented with 0.05% Tween-20 and 0.5% milk, and the membranes were washed as described after both incubations. The membranes were revealed using Clarity ECL Substrate (Bio-Rad) solutions and images were acquired using GE Healthcare Amersham Imager 600. Unprocessed scans of Western Blots are available in the Source Data files. Replicates of western blots for Figs. 1b, 7a, and S6b are available on Figshare (10.6084/m9.figshare.c.6383685.v1).

### LptD antibody production

LptD antibody were produced by CER Marloie (Marloie, Belgium, https://www.cergroupe.be). The custom polyclonal antibody development activities are performed with a commitment to the 3 Rs rules, Replacement, Reduction, and Refinement. To generate polyclonal Ab against LptD for Western blot analysis, the predicted N-terminal soluble part of LptD without signal peptide (nucleotides 109–666 bp from *B. abortus lptD*) was amplified by PCR and cloned into the overexpression vector pET15b generating a N-terminal His_6_ tag fusion (pET15b_lptD). Overexpression was done in *E. coli* BL21(DE3) in the presence of carbenicillin. Bacterial culture was induced with IPTG (1 mM final concentration) for 4 h at 37 °C shaking, lysed by sonication and purified with Ni-NTA resin. The insoluble fraction was further treated in the presence of urea. The soluble and insoluble fractions were analyzed by SDS-PAGE. The protein was present in the insoluble fraction and used for immunization in a rabbit at CER Marloie. 50 μg of antigens with Complete Freund Adjuvant were injected at day 0. Every 4 weeks, 50 μg of antigens with Incomplete Freund Adjuvant were injected until 5 injections in total. Samples of bleeding were taken after 84, 112 and after 120 days for final bleeding. The antisera were tested by Western blot and used at a final dilution of 1:5000.

### IF- Scanning electron microscopy (IF-SEM)

Eighty μl of exponential growth phase bacteria were washed twice with PBS (8200 × *g*., 2.5 min) and fixed with 2% paraformaldehyde (PFA) for 20 min at RT. After two washings with PBS, bacteria were labeled with an Ab directed against the 3Flag (1:20) (Thermo Fisher Scientific) and a secondary Ab anti-mouse IgG conjugated with 18 nm gold particles (1:10) (Abcam). Fifty μg/ml of poly-L-lysine (Sigma-Aldrich) were incubated for 1 h at RT on a coverslip, bacteria were added on coated coverslips and centrifuged at 2342 × *g*. for 5 min. Samples were post-fixed with 200 μl of 2.5% glutaraldehyde in 0.1 M cacodylate buffer pH 7.4 for 1 h at RT. After two washings with 0.2 M cacodylate buffer pH 7.4, cover slips were dehydrated in ethanol with increasing concentrations from 30 to 100% ethanol. Critical Point Drying was done with CPD030 critical point dryer (BALZER) and samples were stored air-sealed at RT upon observation. Immediately before imaging, samples were coated with 5 nm chromium (Quorum Q150T ES, Quorum Technologies) and observed with the field emission scanning electron microscope JEOL JSM-7500 F. Images were acquired with the following settings: 20 μA emission current, 15 kV accelerating voltage and probe current of 9. Isolated bacteria were imaged with a magnification of 40,000× by secondary electron imaging (SEI) and subsequently by detection of low angle backscatter electrons (LABE mode) to visualize the gold particles on the bacterial surface. Further image analysis was carried out with ImageJ^60^.

### SEM image analysis

Images recorded by SEM were analyzed with ImageJ^60^ as followed. For non-dividing bacteria, the length was measured with a straight line and bacteria were divided in the middle in two equal parts by a line at a right angle to the main axis of the bacterium (Fig. S2a). From this midcell line, a straight line measured the distance from the midcell to the gold particle. Bacteria showing a visible constriction site were classified as dividing bacteria. If dividing bacteria were found in nearly straight position, bacteria were divided at the constriction site and the distance of gold articles to the constriction site was measured (Fig. S2b). If dividing bacteria displayed a curved position, the length of both cells (mother and future daughter cell) were measured first by straight lines between both pole and constriction site (Fig. S2c). From this cell length line, a straight line in 90° angle was set to the pole facing the constriction site. Distances were measured from the center of the gold particles towards the line highlighting the constriction site. The ratio between distance of gold particles and length of half bacterium or constriction site, respectively, was calculated for each detected gold particle and results were summarized in 5 categories ranging from 0 (close to the middle or constriction site) to 1 (close to the poles). Given that these measurements were performed on three independent samples, distribution of gold particles on the bacterial surface was statistically analyzed by t-test in comparison to the theoretical frequency of random distribution (20%). In order to analyze the asymmetry between the two sides of the bacterium, the standard deviation for a random insertion was calculated using a binomial distribution based on a probability of occurrence of 0.5 for each side of the bacterium. If the difference of particle number between the two sides of the bacterium exceeds 2 standard deviations, it was considered that particles distribution was asymmetric. To analyze the asymmetry between cell poles of non-dividing bacteria, bacteria ≤1 μm were analyzed.

### Reporting summary

Further information on research design is available in the Nature Portfolio Reporting Summary linked to this article.

## Supplementary information

Supplementary Information

Reporting Summary

## Data Availability

All the data generated in this study have been deposited in the Figshare database at 10.6084/m9.figshare.c.6383685.v1^61,62^. Source data are provided with this paper.

## Source data

Source Data

## References

[1] Beveridge TJ (1999). Structures of gram-negative cell walls and their derived membrane vesicles. J. Bacteriol..

[2] Silhavy TJ, Kahne D, Walker S (2010). The bacterial cell envelope. Cold Spring Harb. Perspect. Biol..

[3] Bertani, B. & Ruiz, N. Function and Biogenesis of Lipopolysaccharides. *EcoSal Plus* **8**10.1128/ecosalplus.ESP-0001-2018 (2018). 10.1128/ecosalplus.esp-0001-2018 PMC6091223 30066669

[4] Zhang G, Meredith TC, Kahne D (2013). On the essentiality of lipopolysaccharide to Gram-negative bacteria. Curr. Opin. Microbiol..

[5] Burman LG, Raichler J, Park JT (1983). Evidence for diffuse growth of the cylindrical portion of the Escherichia coli murein sacculus. J. Bacteriol..

[6] Woldringh CL, Huls P, Pas E, Brakenhoff GJ, Nanninga N (1987). Topography of Peptidoglycan Synthesis during Elongation and Polar Cap Formation in a Cell Division Mutant of Escherichia coli MC4100. Microbiology.

[7] Brown PJ (2012). Polar growth in the Alphaproteobacterial order Rhizobiales. Proc. Natl Acad. Sci. USA.

[8] Krol, E. et al. Tol-Pal System and Rgs Proteins Interact to Promote Unipolar Growth and Cell Division in *Sinorhizobium meliloti*. *mBio* **11**10.1128/mBio.00306-20 (2020). 10.1128/mBio.00306-20 PMC7327166 32605980

[9] Krol E, Stuckenschneider L, Kastle Silva JM, Graumann PL, Becker A (2021). Stable inheritance of Sinorhizobium meliloti cell growth polarity requires an FtsN-like protein and an amidase. Nat. Commun..

[10] Zupan JR, Grangeon R, Robalino-Espinosa JS, Garnica N, Zambryski P (2019). GROWTH POLE RING protein forms a 200-nm-diameter ring structure essential for polar growth and rod shape in Agrobacterium tumefaciens. Proc. Natl Acad. Sci. USA.

[11] Moreno, E. & Moriyón, I. in *The Prokaryotes* Vol. 5 Ch. Chapter 17, 315-456 (Springer, New York, NY, 2006).

[12] Vassen V (2019). Localized incorporation of outer membrane components in the pathogen *Brucella abortus*. EMBO J..

[13] Lapaque N, Moriyon I, Moreno E, Gorvel JP (2005). Brucella lipopolysaccharide acts as a virulence factor. Curr. Opin. Microbiol.

[14] Conde-Alvarez R (2012). The lipopolysaccharide core of Brucella abortus acts as a shield against innate immunity recognition. PLoS Pathog..

[15] Bonifer C, Glaubitz C (2021). MsbA: an ABC transporter paradigm. Biochem Soc. Trans..

[16] Caroff M, Bundle DR, Perry MB, Cherwonogrodzky JW, Duncan JR (1984). Antigenic S-type lipopolysaccharide of Brucella abortus 1119-3. Infect. Immun..

[17] Wattam AR (2012). Comparative genomics of early-diverging Brucella strains reveals a novel lipopolysaccharide biosynthesis pathway. mBio.

[18] Dubray G, Limet J (1987). Evidence of heterogeneity of lipopolysaccharides among Brucella biovars in relation to A and M specificities. Annales de. l’Institut Pasteur / Microbiologie.

[19] Bowden RA, Cloeckaert A, Zygmunt MS, Bernard S, Dubray G (1995). Surface exposure of outer membrane protein and lipopolysaccharide epitopes in *Brucella* species studied by enzyme-linked immunosorbent assay and flow cytometry. Infect. Immun..

[20] Whitfield C, Trent MS (2014). Biosynthesis and export of bacterial lipopolysaccharides. Annu. Rev. Biochem..

[21] Godfroid F (2000). Genetic organisation of the lipopolysaccharide O-antigen biosynthesis region of Brucella melitensis 16M (wbk). Res. Microbiol..

[22] Bi Y, Mann E, Whitfield C, Zimmer J (2018). Architecture of a channel-forming O-antigen polysaccharide ABC transporter. Nature.

[23] Owens TW (2019). Structural basis of unidirectional export of lipopolysaccharide to the cell surface. Nature.

[24] Bishop RE (2019). Ratcheting up lipopolysaccharide transport. Nature.

[25] Sternon JF (2018). Transposon Sequencing of *Brucella abortus* Uncovers Essential Genes for Growth In Vitro and Inside Macrophages. Infect. Immun..

[26] Wu T (2006). Identification of a protein complex that assembles lipopolysaccharide in the outer membrane of Escherichia coli. Proc. Natl Acad. Sci. USA.

[27] Botte M (2022). Cryo-EM structures of a LptDE transporter in complex with Pro-macrobodies offer insight into lipopolysaccharide translocation. Nat. Commun..

[28] Benn G (2021). Phase separation in the outer membrane of Escherichia coli. Proc. Natl Acad. Sci. USA.

[29] Hallez R (2007). The asymmetric distribution of the essential histidine kinase PdhS indicates a differentiation event in Brucella abortus. EMBO J..

[30] Narita S, Tokuda H (2009). Biochemical characterization of an ABC transporter LptBFGC complex required for the outer membrane sorting of lipopolysaccharides. FEBS Lett..

[31] Kuru E (2012). In Situ probing of newly synthesized peptidoglycan in live bacteria with fluorescent D-amino acids. Angew. Chem. Int Ed. Engl..

[32] Deghelt M (2014). G1-arrested newborn cells are the predominant infectious form of the pathogen *Brucella abortus*. Nat. Commun..

[33] Orelle C, Dalmas O, Gros P, Di Pietro A, Jault JM (2003). The conserved glutamate residue adjacent to the Walker-B motif is the catalytic base for ATP hydrolysis in the ATP-binding cassette transporter BmrA. J. Biol. Chem..

[34] Li H (2017). The redefinition of Helicobacter pylori lipopolysaccharide O-antigen and core-oligosaccharide domains. PLoS Pathog..

[35] Perez-Burgos M, Garcia-Romero I, Jung J, Valvano MA, Sogaard-Andersen L (2019). Identification of the lipopolysaccharide O-antigen biosynthesis priming enzyme and the O-antigen ligase in Myxococcus xanthus: critical role of LPS O-antigen in motility and development. Mol. Microbiol..

[36] Schild S, Lamprecht AK, Reidl J (2005). Molecular and functional characterization of O antigen transfer in Vibrio cholerae. J. Biol. Chem..

[37] Islam ST, Taylor VL, Qi M, Lam JS (2010). Membrane topology mapping of the O-antigen flippase (Wzx), polymerase (Wzy), and ligase (WaaL) from Pseudomonas aeruginosa PAO1 reveals novel domain architectures. mBio.

[38] Varadi M (2022). AlphaFold Protein Structure Database: massively expanding the structural coverage of protein-sequence space with high-accuracy models. Nucleic Acids Res..

[39] Jumper J (2021). Highly accurate protein structure prediction with AlphaFold. Nature.

[40] Perez JM, McGarry MA, Marolda CL, Valvano MA (2008). Functional analysis of the large periplasmic loop of the Escherichia coli K-12 WaaL O-antigen ligase. Mol. Microbiol..

[41] Ashraf KU (2022). Structural basis of lipopolysaccharide maturation by the O-antigen ligase. Nature.

[42] Gonzalez D (2008). Brucellosis vaccines: assessment of Brucella melitensis lipopolysaccharide rough mutants defective in core and O-polysaccharide synthesis and export. PLoS One.

[43] Vizcaino N, Cloeckaert A, Zygmunt MS, Fernandez-Lago L (1999). Molecular characterization of a Brucella species large DNA fragment deleted in Brucella abortus strains: evidence for a locus involved in the synthesis of a polysaccharide. Infect. Immun..

[44] Vizcaino N, Verger JM, Grayon M, Zygmunt MS, Cloeckaert A (1997). DNA polymorphism at the omp-31 locus of Brucella spp.: evidence for a large deletion in Brucella abortus, and other species-specific markers. Microbiol. (Read.).

[45] Salvador-Bescos M (2018). WadD, a New Brucella Lipopolysaccharide Core Glycosyltransferase Identified by Genomic Search and Phenotypic Characterization. Front. Microbiol..

[46] Ruan X, Loyola DE, Marolda CL, Perez-Donoso JM, Valvano MA (2012). The WaaL O-antigen lipopolysaccharide ligase has features in common with metal ion-independent inverting glycosyltransferases. Glycobiology.

[47] Jorgenson MA, Young KD (2016). Interrupting Biosynthesis of O Antigen or the Lipopolysaccharide Core Produces Morphological Defects in Escherichia coli by Sequestering Undecaprenyl Phosphate. J. Bacteriol..

[48] Godessart P (2021). beta-Barrels covalently link peptidoglycan and the outer membrane in the alpha-proteobacterium *Brucella abortus*. Nat. Microbiol..

[49] Mamou G (2022). Peptidoglycan maturation controls outer membrane protein assembly. Nature.

[50] Kumar M, Mommer MS, Sourjik V (2010). Mobility of cytoplasmic, membrane, and DNA-binding proteins in Escherichia coli. Biophys. J..

[51] Han W (2012). Defining function of lipopolysaccharide O-antigen ligase WaaL using chemoenzymatically synthesized substrates. J. Biol. Chem..

[52] Grabowicz M (2014). A mutant Escherichia coli that attaches peptidoglycan to lipopolysaccharide and displays cell wall on its surface. Elife.

[53] Cloeckaert A, Grayon M, Verger J-M, Letesson J-J, Godfroid F (2000). Conservation of seven genes involved in the biosynthesis of the lipopolysaccharide O-side chain in Brucella spp. Res. Microbiol..

[54] Porte F, Naroeni A, Ouahrani-Bettache S, Liautard JP (2003). Role of the Brucella suis lipopolysaccharide O antigen in phagosomal genesis and in inhibition of phagosome-lysosome fusion in murine macrophages. Infect. Immun..

[55] Godfroid F (1998). Identification of the perosamine synthetase gene of Brucella melitensis 16M and involvement of lipopolysaccharide O side chain in Brucella survival in mice and in macrophages. Infect. Immun..

[56] Price RE, Templeton JW, Adams LG (1990). Survival of smooth, rough and transposon mutant strains of Brucella abortus in bovine mammary macrophages. Vet. Immunol. Immunopathol..

[57] Allen CA, Adams LG, Ficht TA (1998). Transposon-derived Brucella abortus rough mutants are attenuated and exhibit reduced intracellular survival. Infect. Immun..

[58] Schindelin J (2012). Fiji: an open-source platform for biological-image analysis. Nat. Methods.

[59] Ducret A, Quardokus EM, Brun YV (2016). MicrobeJ, a tool for high throughput bacterial cell detection and quantitative analysis. Nat. Microbiol..

[60] Schneider, C. A. Rasband, W. S. & Eliceiri, K. W. NIH Image to ImageJ: 25 years of image analysis. *Nat Methods* **9**, 671-675 (2012). 10.1038/nmeth.2089 PMC5554542 22930834

[61] Servais, C. & De Bolle, X. Source data for Lipopolysaccharide biosynthesis and traffic in the envelope of the pathogen Brucella abortus. 10.6084/m9.figshare.c.6383685.v1 (2023). 10.1038/s41467-023-36442-y PMC9938171 36806059

[62] Waterhouse A (2018). SWISS-MODEL: homology modelling of protein structures and complexes. Nucleic Acids Res..

[63] Boratyn GM (2012). Domain enhanced lookup time accelerated BLAST. Biol. Direct.

[64] Goujon M (2010). A new bioinformatics analysis tools framework at EMBL-EBI. Nucleic Acids Res..

[65] Sievers F (2011). Fast, scalable generation of high-quality protein multiple sequence alignments using Clustal Omega. Mol. Syst. Biol..

[66] Hallgren, J. et al. DeepTMHMM predicts alpha and beta transmembrane proteins using deep neural networks. *BioRxiv*10.1101/2022.04.08.487609 (2022).

[67] Schrodinger, LLC. The PyMOL Molecular Graphics System, Version 1.8 (2015).

