# Supplementary material for: Lipopolysaccharide biosynthesis and traffic in the envelope of the pathogen Brucella abortus
Source: Nat Commun. 2023 Feb 17;14:911. doi: 10.1038/s41467-023-36442-y (PMC9938171; doi:10.1038/s41467-023-36442-y)
Supplement: Supplementary file 1 — Supplementary Information [file 41467_2023_36442_MOESM1_ESM.pdf]

Supplementary Information

For Servais *et al,* "Lipopolysaccharide synthesis and traffic in the envelope of the pathogen *Brucella abortus*"

Table S1 | List of strains used in this study

Table S2 | List of primers used in this study

Table S3 | List of plasmids used in this study

Table S4 | List of primary antibodies used in this study

Table S5 | List of secondary antibodies used in this study

Table S6 | Table of ORF investigated in this study

Table S7 | Table representing the percentage of S-LPS and LptCF protein colocalization upon S-LPS induction

Figure S1 | Alignment of Rhizobiales LptD amino acid sequences

Figure S2 | Localization of LptD

Figure S3 | Growth behaviour of *mNG-lptC*, *mNG-lptF* and *msbA-mNG* strains

Figure S4 | Polar localization of Rhizobiales growth and septation protein RgsE is conserved in *B. abortus*

Figure S5 | MsbA localization in *B. abortus*

Figure S6 | S-LPS phenotype of *B. abortus* Δ*waaL*

Figure S7 | S-LPS labelling of *B. melitensis* 16M strains

Figure S8 | Wzm appears dispersed surrounding the bacterial cell

Table S1 | List of strains used in this study.

Name	Genotype	Reference
	Brucella abortus 544 strains	
**Wild type (WT)**	*B. abortus* 544, Nal^R	J.-M.Verger, INRA, Tours
rgsE-mNG pdhS-mCherry	*B. abortus* 544 *rgsE-mNG pdhS*::pSK kan *pdhs-mCherry*	This study
lptD-AGDP	*B. abortus* 544 *lptD-AGDP-G4-3Flag*	V. Vassen, PhD thesis, UNamur, BE
mNG-lptC pdhS-mCherry	*B. abortus* 544 *mNG-lptC pdhS*::pSK kan *pdhs-mCherry*	This study
mNG-lptF pdhS-mCherry	*B. abortus* 544 *mNG-lptF pdhS*::pSK kan *pdhs-mCherry*	This study
msbA-mNG pdhS-mCherry	*B. abortus* 544 *msbA-mNG pdhS*::pSK kan *pdhs-mCherry*	This study
**pSK_*msbAmNG pKS popZ-mCherry***	*B. abortus 544 msbA::pSK kan msbA-mNG popZ::pSK kan popZ-mCherry*	This study
**pSK_*$msbA_{E491A}$-mNG pKS popZ-mCherry***	*B. abortus* 544 *msbA*::pSK kan *$msbA_{E491A}$-mNG popZ*::pSK kan *popZ-mCherry*	This study
Δgmd	*B. abortus* 544 Δ*gmd*	[1]
ΔwaaL	*B. abortus* 544 Δ*waaL*	This study
ΔgmdΔwadA	*B. abortus* 544 Δ*gmd*Δ*wadA*	This study
ΔgmdΔOAg-lig	*B. abortus* 544 Δ*gmd*Δ*OAg-lig*	This study
Δgmd* pBBRI *gmd	*B. abortus* 544 Δ*gmd* pBBRI_*gmd*	This study
ΔgmdΔwadA* pBBRI *gmd	*B. abortus* 544Δ*gmd*Δ*wadA* pBBRI_*gmd*	This study
ΔgmdΔOAg-lig* pBBRI *gmd	*B. abortus* 544 Δ*gmd*Δ*OAg-lig* pBBRI_*gmd*	This study
mNG-wadA	*B. abortus* 544 *mNG-wadA*	This study
wadA-mNG	*B. abortus* 544 *wadA-mNG*	This study
mNG-wzm	*B. abortus* 544 *mNG-wzm*	This study
	Brucella melitensis 16M	
**Wild type (WT)**	*B. melitensis* 16M, Nal^R	A. Macmillan, Central Veterinary Laboratory, Weybridge, UK
Δgmd	*B. melitensis* 16M Δ*gmd*	This study
ΔwadA	*B. melitensis* 16M Δ*wadA*	This study
ΔOAg-lig	*B. melitensis* 16M Δ*OAg-lig*	This study
ΔwadA* pBBR *wadA	*B. melitensis* 16M Δ*wadA* pBBR_MCSI_*wadA*	This study
ΔOAg-lig* pBBR *OAg-lig	*B. melitensis* 16M Δ*OAg-lig* pBBR_MCSI_*OAg-lig*	This study
ΔwadA* pBBR *wadA-R614A	*B. melitensis* 16M Δ*wadA* pBBR_MCSI_*wadA-R614A*	This study

	Agrobacterium tumefaciens C58	
**WT**	*A. tumefaciens* C58, Amp^R	Strain CFBP #1903 ATCC33970
mNG-lptC	*A. tumefaciens* C58 *mNG-lptC*	This study
mNG-lptF	*A. tumefaciens* C58 *mNG-lptF*	This study

Table S2 | List of primers used in this study. Nucleotides not annealing on DNA template are shown in bold, red characters represent point mutations and UPPERCASE corresponds to restriction site.

Primer name	5'-3' sequence
**pNPTS138-rgsEmNG-F**	**gagcccggcgcagagctgac**atctggatccacgaattcgc
**pNPTS138-rgsEmNG-R**	**ttcagcaagttccttgagcg**atcctgcagagaagcttggc
**rgsEmNG-UP-F**	**gccaagcttctctgcaggat**cgctcaaggaacttgctgaaattgt
**rgsEmNG-UP-R**	**tcttcttcgcccttcgagac**ccgaaggcgtccgcttgcat
**mNG-4-rgsE-F**	**atgcaagcggacgccttcgg**gtctcgaagggcgaagaaga
**mNG-4-rgsE-R**	**ttttaactggagccgctcta**cttatacagttcatccatgccc
**rgsEmNG-DW-F**	**gcatggatgaactgtataag**tagagcggctccagttaaaattgagca
**rgsEmNG-DW-R**	**gcgaattcgtggatccagat**gtcagctctgcgccgggctc
**check-rgsEmNG-F**	aagcgaaggaaacgacgac
**check-rgsEmNG-R**	acttgcaattgctgatccc
**lptD_AGDP_UP_F_SpeI**	**ctagaACTAGT**gaaatcaccacattcaacaatgg
**lptD_AGDP-G4-3F-4_UP-R2**	**cgatgtcatgatctttataatcaccgtcatggtctttgtagtcgccaccgccgcc**cgg atcgcccgccaggtag
***lptD*_AGDP-G4-3F-G4_DW-F**	**ttataaagatcatgacatcgattacaaggatgacgatgacaagggcggcggtggc**aatga aatgtattcaaagcagc
***lptD*_AGDP_DW-R_SalI**	**tgcac**GTCGACgccaagctggaaggactg
**mNGlptC-UP-F**	caggatgacacccttcac
**mNGlptC-UP-R**	**tcatggtctttgtagtccat**gttatcctttggcacatca
**mNG-4-lptC-F**	atggactacaaagaccatga
**mNG-4-lptC-R**	cggaacatgaatatgtggta
**mNGlptC-DW-F**	**cggtcggccaccggatcc**acggccgacataccacat
**mNGlptC-DW-R**	ttgtttgccgaaagcgtg
**check-mNGlptC-F**	aaaagggtcatgccacg
**check-mNGlptC-R**	tcgtgcatgtctttatccc
**mNGlptF-UP-F**	ggcagtcttgcccaaag
**mNGlptF-UP-R**	**gtcatggtctttgtagtccat**aggtggagtctacatccc
**mNG-4-lptF-F**	atggactacaaagaccatga
**mNG-4-lptF-R**	**aaaTCTAGA**cttatacagttcatccatgc
**mNGlptF-DW-F**	**aaaTCTAGAcggtcggccaccggatcc**cgattgatcgagttgtaca
**mNGlptF-DW-R**	ttttccatcagcagcatg
**check-mNGlptF-F**	ttcctctctcatgtgaga
**check-mNGlptF-R**	ccgaatcctgattcatcg
**pNPTS-mNG-lptC-AT-F**	**gcgaagttccagtctgcgga**attcgctagcttcggccgtg

**pNPTS-mNG-lptC-AT-R**	**cgcttccgcaggttatatga**tcgtggatccagatatcctgc
**mNGlptC-AT-UP-F**	**caggatatctggatccacga**tcatataacctgcggaagcg
**mNGlptC-AT-UP-R**	**tcttcgcccttcgagaccac**aatcgaagcttgtgtctcta
**mNG-4-lptC-AT-F**	**tagagacacaagcttcgatt**gtggtctcgaagggcgaaga
**mNG-4-lptC-AT-R**	**cgggatccggtggccgaccg**cttatacagttcatccatgccca
**mNGlptC-AT-DW-F**	**agcggtcggccaccggatcc**cgcctgaatggcgccgggga
**mNGlptC-AT-DW-R**	**cacggccgaagctagcgaat**tccgcagactggaacttcgcttgt
**Check-mNGlptC-AT-F**	**tcccttgcacctctccag**
**Check-mNGlptC-AT-R**	**cagaacacgaccagcagc**
**pNPTS-mNG-lptF-AT-F**	**tgccaaggaaggcatgatcg**attcgctagcttcggccgtg
**pNPTS-mNG-lptF-AT-R**	**gcggctgcccatttcaagaa**tcgtggatccagatatcctgc
**mNGlptF-AT-UP-F**	**caggatatctggatccacga**ttcttgaaatgggcagccgc
**mNGlptF-AT-UP-R**	**tcttcgcccttcgagaccat**acggccctgttcgatattgt
**mNG-4-lptF-AT-F**	**acaatatcgaacagggccgt**atggtctcgaagggcgaaga
**mNG-4-lptF-AT-R**	**ttggatccggtggccgaccg**cttatacagttcatccatgccc
**mNGlptF-AT-DW-F**	**agcggtcggccaccggatcc**aagcttctcgagaactatatcct
**mNGlptF-AT-DW-R**	**cacggccgaagctagcgaat**cgatcatgccttccttggca
**Check-mNGlptF-AT-F**	**ccaattgccaacgatgcg**
**Check-mNGlptF-AT-R**	**acgaaccagatggtcgtc**
**msbAmNG-UP-F**	ttctatgacctcgaccgg
**msbAmNG-UP-R**	**ggatccggtggccgaccg**tctggcctctcctgtg
**mNG-4-msbAm-F2**	**cggtcggccaccggatcc**gactacaaagaccatgacg
**mNG-4-msbAm-R2**	**cacttcctcgctca**cttatacagttcatccatgcc
**msbAmNG-DW-F**	tgagcgaggaagtgcaa
**msbAmNG-DW-R**	ggccagtatagccatcac
**check-msbAmNG-F**	tgcgcaacctctatctg
**check-msbAmNG-R**	ggccagtatagccatcac
**pSKmsbAmNG-F**	**aaaACTAGT**tgcgcaacctctatctg
**pSKmsbAmNG-R**	**ttttTCTAGA**tcacttatacagttcatccatg
**pSKmsbA$_{E491A}$-mNG-UP-F**	**aaaACTAGT**tgcgcaacctctatctggg
**pSKmsbA$_{E491A}$-mNG-UP-R**	**ttgtcgagcgcggacgttgctg**cgtcgagaagcaggaccg
**pSKmsbA$_{E491A}$-mNG-DW-F**	tcgacg**ca**gc**a**acgtcc
**pSK msbA$_{E491A}$-mNG-DW-R**	**tttTCTAGA**tcacttatacagttcatccatgccc
**check-Δgmd-F**	agctaacttgctggcataag
**check-Δgmd R**	caggtcgagtcgatcatg
**ΔwaaL-UP-F**	aattgcaatgcctataatgc
**ΔwaaL-UP-R**	**ttcatgaagtcactccga**tcagatatccaaaatgtgcc
**ΔwaaL-DW-F**	tcggagtgacttcatgaa
**ΔwaaL-DW-R**	cgccatatgcagatgttcg
**ΔwadA-UP-F**	gatcaagcaggtgctcatc
**ΔwadA-UP-R**	**tgtaaaagtggaaccgctc**taattcgcttgcctcagc

**ΔwadA-DW-F**	gagcggttccacttttaca
**ΔwadA-DW-R**	gcaaatatcagcgcaacg
**check-ΔwadA-F**	gaaacggtgcggataatc
**check-ΔwadA-R**	aacgcatatcgaagcttt
**ΔOAg-lig-UP-F**	cgaggttcgcatatatctatc
**ΔOAg-lig-UP-R**	taacaattcccaggatgtatt
**ΔOAg-lig-DW-F**	**aatacatcctgggaattgtta**attcgctatggatttctgg
**ΔOAg-lig-DW-R**	gaagtgctggcaaacttt
**check-ΔOAg-lig-F**	gaaacggtgcggataatc
**check-ΔOAg-lig-R**	aacgcatatcgaagcttt
**pBBRI-wadAc-F**	**tttCTGCAG**atttctcgctcgatcgc
**pBBRI-wadAc-R**	**tttCTCGAG**attacaggcttgtcaggg
**pBBRI-OAg-lig-UP-F**	**ttttGAGCTC**atttctcgctcgatcgc
**pBBRI-OAg-lig-UP-R**	**aaaaCATATG**cattaattcgcttgcctca
**pBBRI-OAg-lig-DW-F**	**aaaCATATG**tccgatcagaattcaggtt
**pBBRI-OAg-lig-DW-R**	**aaaaCTGCAG**attacaggcttgtcaggg
**pBBRwadA-R614A-UP-F**	**tttCTGCAG**atttctcgctcgatcgc
**pBBRwadA-R614A-UP-R**	catag**gc**aatggcaatttccag
**pBBRwadA-R614A-DW-F**	**ctggaaattgccattgcctatg**gatttctgggcttgctgt
**pBBRwadA-R614A-DW-F**	**tttCTCGAG**attacaggcttgtcaggg
**mNG-wadA-UP-F**	tcaaccttctctccaatgc
**mNG-wadA-UP-R**	**cgtcatggtctttgtagtc**cattaattcgcttgcctc
**mNG4wadA-F**	gactacaaagaccatgacg
**mNG4wadA-R**	**ggatccggtggccgaccg**cttatacagttcatccatgc
**mNG-wadA-DW-F**	**cggtcggccaccggatcc**atattgcccgtattcgtca
**mNG-wadA-DW-R**	tttgagatttcgctgtgag
**wadA-mNG-UP-F**	aaaagccagacctcacg
**wadA-mNG-UP-R**	**ggatccggtggccgaccg**gaagtaggtgcgcggg
**mNG4wadAm-F**	**cggtcggccaccggatcc**gactacaaagaccatgacggt
**mNG4wadAm-R**	**taaaagtggaaccgctctta**cttatacagttcatccatgccc
**wadA-mNG-DW-F**	taagagcggttccactttta
**wadA-mNG-DW-R**	acctgggaacatatggcg
**Check-wadA-F**	gatcaagcaggtgctcatc
**Check-wadA-R**	gcaaatatcagcgcaacg
**pNPTS138_mNG-wzm-F**	**gagccatttttacctatga**atctggatccacgaattcgc
**pNPTS138_mNG-wzm-R**	**cgatttttcgtcacaaatct**atcctgcagagaagcttggc
**mNG-wzm-UP-F**	**gccaagcttctctgcaggat**agatttgtgacgaaaaatcgc
**mNG-wzm-UP-R**	**tcttcttcgcccttcgagac**catacgaaatcgtctcactaaa
**mNG4wzm-F**	**tagtgagacgatttcgtatg**gtctcgaagggcgaagaaga
**mNG4wzm-R**	**acattagccatatacgatat**cttatacagttcatccatgccc
**mNG-wzm-DW-F**	**gcatggatgaactgtataag**atatcgtatatggctaatgtctgg

**mNG-wzm-DW-R**	**gcgaattcgtggatccagat**tcataggtaaaaaatggctctc
**Check-mNG-wzm**	tgtgacatggattcgatcc
**Check-mNG-wzm**	cagggtaatcgatggctg

Table S3 | List of plasmids used in this study.

Plasmid name	Reference
**pNPTS138**	M. R. K. Alley, Imperial College of Science,London, UK
**pSKoriT kan**	[2]
**pKSoriT cat**	Isabelle Danese and Pascal Lestrate, UNamur, BE
**pBBR_MCSI**	[3]
**pNPTS138_*rgsE-mNG***	This study
**pNPTS138 *lptD::lptD-3Flag***	V. Vassen, PhD thesis, UNamur, BE
**pNPTS138_*mNG-lptC***	This study
**pNPTS138_*mNG-lptF***	This study
**pNPTS138_*msbA-mNG***	This study
**pSKoriT_kan_*pdhS-mCherry***	[4]
**pKSoriT_cat_*popZ-mCherry***	[5]
**pSKoriT_kan-*msbA-mNG***	This study
**pSKoriT_kan- $msbA_{E491A}$-*mNG***	This study
**pNPTS138_*mNG-lptC-AT***	This study
**pNPTS138_*mNG-lptF-AT***	This study
**pNPTS138_Δ*waaL***	This study
**pNPTS138_Δ*wadA***	This study
**pNPTS138_Δ*OAg-lig***	This study
**pBBR_MCSI_*waaL***	This study
**pBBR_MCSI_*wadA***	This study
**pBBR_MCSI_*OAg-lig***	This study
**pBBR_MCSI_*wadA-R614A***	This study
**pBBRI_P_{lacZ}_*gmd***	[1]
**pNPTS138_Δ*gmd***	[1]
**pNPTS138_*mNG_wadA***	This study
**pNPTS138_*wadA_mNG***	This study
**pNPTS138_*mNG_wzm***	This study

Table S4 | List of primary antibodies used in this study.

Name	Recognized structure	Host (Isotype)	Application	Reference
**A76/12G12**	*Brucella* S-LPS (O-antigen)	Mouse (IgG1)	WB, IF	[6]
**B66/04F09**	*Brucella* S-LPS (O-antigen)	Mouse (IgG2a)	IF, S-LPS induction	[7]

**A68/24D08/G09**	*Brucella* R-LPS (lateral branch of the core)	Mouse (Unknown)	WB	[8]
**Anti-LptD**	*Brucella* LptD	Rabbit (Polyclonal)	WB	This study
**DYKDDDDK Tag (FG4R)**	3Flag tag	Mouse (IgG2b)	WB, IF- SEM	ThermoFisher Scientific
**A68/7G11/C10**	*Brucella* Omp10	Mouse (IgG2a)	WB	[9]

Table S5 | List of secondary antibodies used in this study.

Target	Conjugate	Application	Reference
**Mouse IgG (H+L)**	Alexa Fluor 514	IF	Invitrogen (A-31555)
**Mouse**	HRP	WB	Dako (P0260)
**Rabbit**	HRP	WB	Dako (P0217)
**Mouse**	18 nm gold	IF-SEM	Abcam

Table S6 | Table of ORF investigated in this study.

**Gene name**	**ORF number in *B. abortus* 2308**	**UniProt accession number**	**NCBI Hyperlink**
rgsE	BAB1_1043	Q2YQ47	https://www.ncbi.nlm.nih.gov/protein/CAJ10999.1/
lptD	BAB1_0707	Q2YN48	https://www.ncbi.nlm.nih.gov/protein/CAJ10663.1/
lptC	BAB1_0154	Q2YP16	https://www.ncbi.nlm.nih.gov/protein/CAJ10110.1/
lptF	BAB1_0709	Q2YN46	https://www.ncbi.nlm.nih.gov/protein/CAJ10665.1/
msbA	BAB2_1011	Q2YJN8	https://www.ncbi.nlm.nih.gov/protein/CAJ13177.1/
gmd	BAB1_0545	Q2YMP3	https://www.ncbi.nlm.nih.gov/protein/CAJ10501.1/
waaL	BAB2_0106	Q2YJ92	https://www.ncbi.nlm.nih.gov/protein/CAJ12272.1/
wadA	BAB1_0639	Q2YMX2	https://www.ncbi.nlm.nih.gov/protein/CAJ10595.1/
wzm	BAB1_0543	Q2YMP5	https://www.ncbi.nlm.nih.gov/protein/CAJ10499.1/
**Gene name**	**ORF in *A. tumefaciens* C58**	**UniProt accession number**	**NCBI Hyperlink**
lptC	Atu0335	Q7D1N8	https://www.ncbi.nlm.nih.gov/protein/15155241
lptF	Atu1108	A9CJH0	https://www.ncbi.nlm.nih.gov/protein/15156140

Table S7 | Table representing the percentage of S-LPS and LptCF protein colocalization upon S-LPS induction. Only the bacteria displaying focus for LptC/F and labelling for S-LPS were taken into account for the analysis. When S-LPS signal was detected at one pole, LptC and LptF were also detected at the same pole in 67.18 and 77.70% of the case. In 9.06 and 2.59% of bacteria for mNG-lptC or mNG-lptF respectively, no signal was detected for S-LPS when a focus could be observed at the mid cell either for LptC or LptF. This could be due to a low amount of newly incorporated S-LPS at the moment of the labelling that would be below the detection threshold. In 7.86 and 4.60% of the time, LptC and LptF were observed at both poles of the bacteria when the S-LPS signal was only at one pole. LptC and LptF were sometimes (in 3.76 and 6.61% respectively) localized at the opposite pole to the one labelled for S-LPS. Mid cell localisation was also observed in 1.88% for LptC and 2.30% for LptF for bacteria only labelled at one pole for S-LPS. When the S-LPS signal was also detected at the new pole and the constriction site, LptC and LptF foci could be either detected at the same area as S-LPS (4.79% and 2.73% respectively), or at the mid cell only (4.96 and 5.47% respectively). Lastly, S-LPS signal was sometimes observed at the mid cell only, and in that case, 0.51% of the bacteria also displayed a focus for LptC, and 1% of the bacteria displayed a focus for LptF When the signals are not colocalizing, the percentage is represented in grey. Although not represented here, it should be stated that after 9h of IPTG induction, only 20% and 16% of the whole bacterial population were labelled for S-LPS incorporation in *mNG-lptC* and *mNG-lptF,* as opposed to about 41%for the control (Δ*gmd* pBBRi *gmd*). Therefore, one should note that even if the fusions (*mNG-lptC* and *mNG-lptF*) are viable, the functionality of the fusion proteins might slightly impair the efficacy of LPS export to the OM.

	**% of LptC foci**	**% of LptF foci**
**S-LPS signal at one pole**		
Lpt protein at the same pole	67.18	77.70
Lpt protein at the same pole & at mid cell	9.06	2.59
Lpt protein bipolar	7.86	4.61
Lpt protein opposite pole	3.76	3.60
Lpt protein at mid cell	1.88	2.30
**S-LPS signal at one pole and at the mid cell**		
Lpt protein at same pole & at mid cell	4.79	2.73
Lpt protein at mid cell	4.96	5.47
**S-LPS signal at mid cell**		
Lpt protein at mid cell	0.51	1.00
Total	100	100

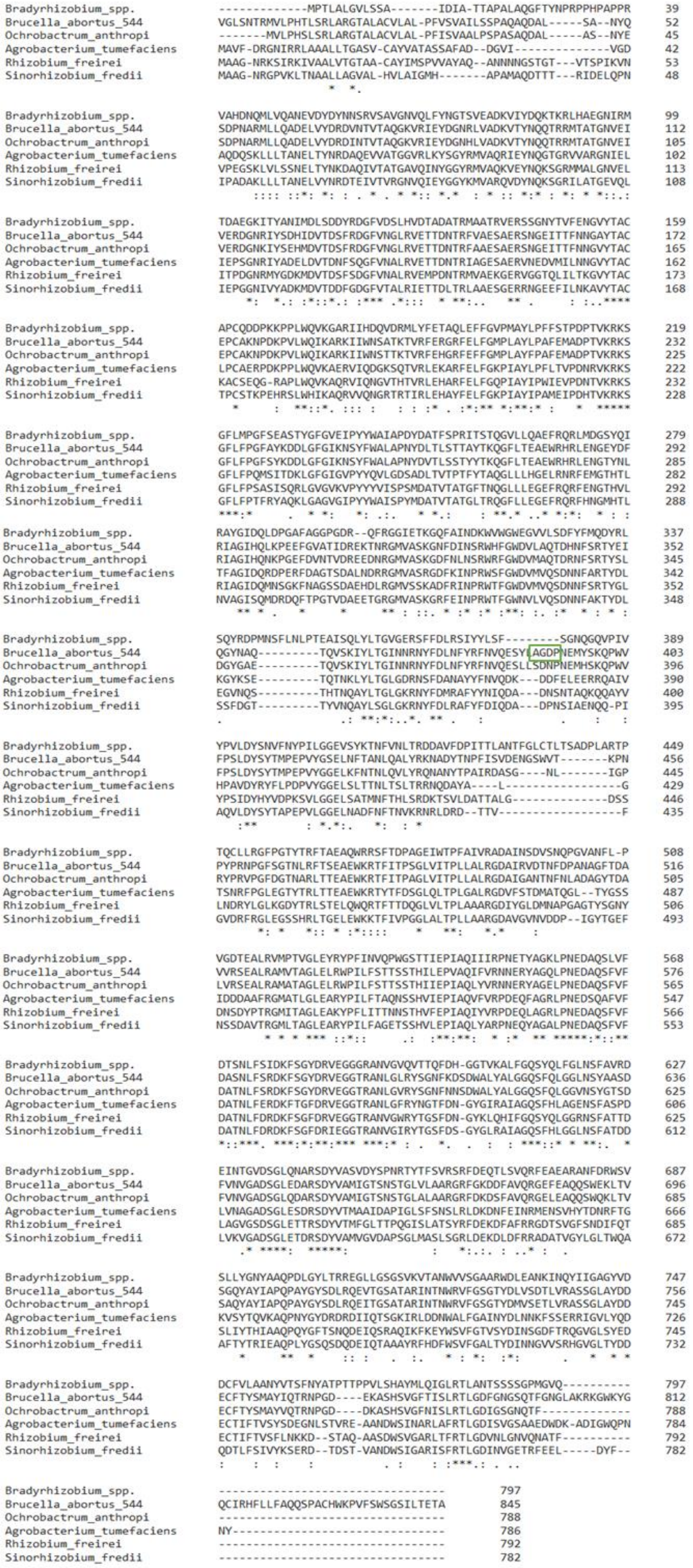

Figure S1 | Alignment of Rhizobiales LptD amino acid sequences. Sequences of LptD homologs in other Rhizobiales were identified by Blast-P [10] and multiple sequence alignment was generated with CLUSTAL Omega (1.2.4) [11]. Position of the insertion of a 3Flag tag is shown with a green box (AGDP loop, position 383 in *B. abortus* 544 sequence). The ADGP sequence was duplicated and a Gly_4 linker sequence was inserted on each side of the 3Flag.

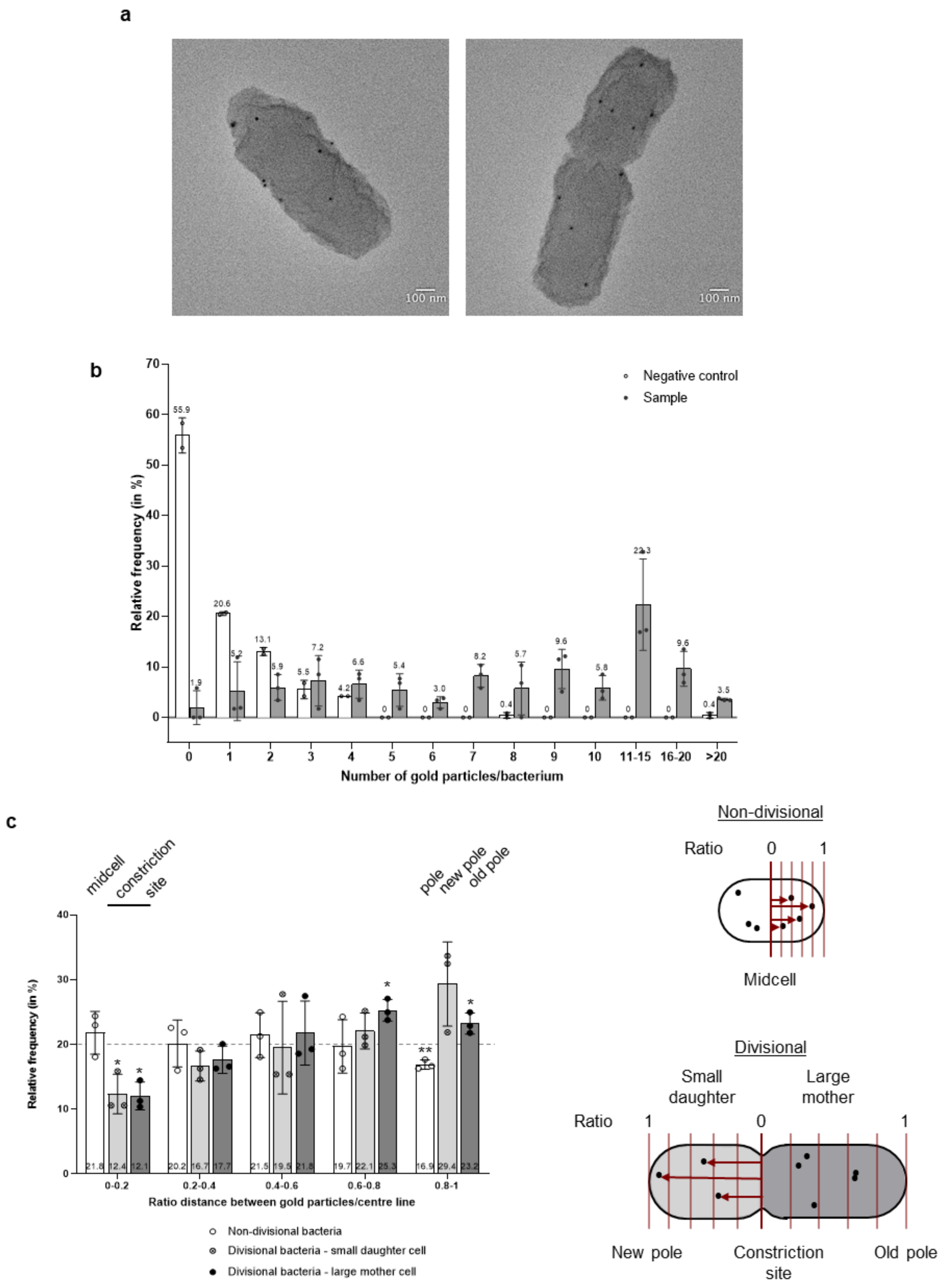

Figure S2 | Localization of LptD. a, SEM images from exponential phase culture of *B. abortus* WT and *3Flag::lptD*. Scale bar is 100 nm. Images were acquired by low angle backscattered electron (LABE) mode and inverted. Black dots correspond to gold particles. Brightness (+20%) and sharpness (+50%) of images were adjusted (n=3). **b,** Frequency distribution of gold particles from negative control (*B. abortus* disrupt *gmd*) and sample (*B. abortus* 544 disrupt *gmd lptD*-3Flag) after IF-SEM. Data are presented as mean values (corresponding numbers are shown on top of the bars) and error bars correspond to standard deviation from two and three independent experiments, respectively. $n_{negative\ control}$= 142 bacteria, n_{sample}=169 bacteria. Source data are provided as a Source Data file**. c,** The distribution of gold particles labelling 3Flag::LptD was analyzed by measuring the distance towards central line in non-divisional and towards constriction site in divisional bacteria. Ratios of distance of gold particles/central line were classified in five categories of equal size (model on right side, ratio distances as shown as red arrows). Divisional bacteria were further divided in daughter and mother part according to their size. Relative frequencies are shown as mean values (corresponding numbers are shown at the bottom of each bar) and error bars correspond to standard deviation from three independent experiments. Grey dashed line represents theoretical frequency of random distribution (20%). Statistical differences compared to a theoretical relative frequency of 20% were analyzed by one-sided t-test. **p*<0.05 (0.025 for small daughter (ratio of 0-0.2), 0,012 for large mother cell (ratio 0-0.2), 0.016 for large mother cell (ratio 0.6-0.8), and 0.016 for large bacteria (ratio 0.8-1)), ***p*<0.01 (non-divisional bacteria (ratio 0.8-1)). Number of non-divisional bacteria= 58, Number of divisional bacteria=77. Source data are provided as a Source Data file.

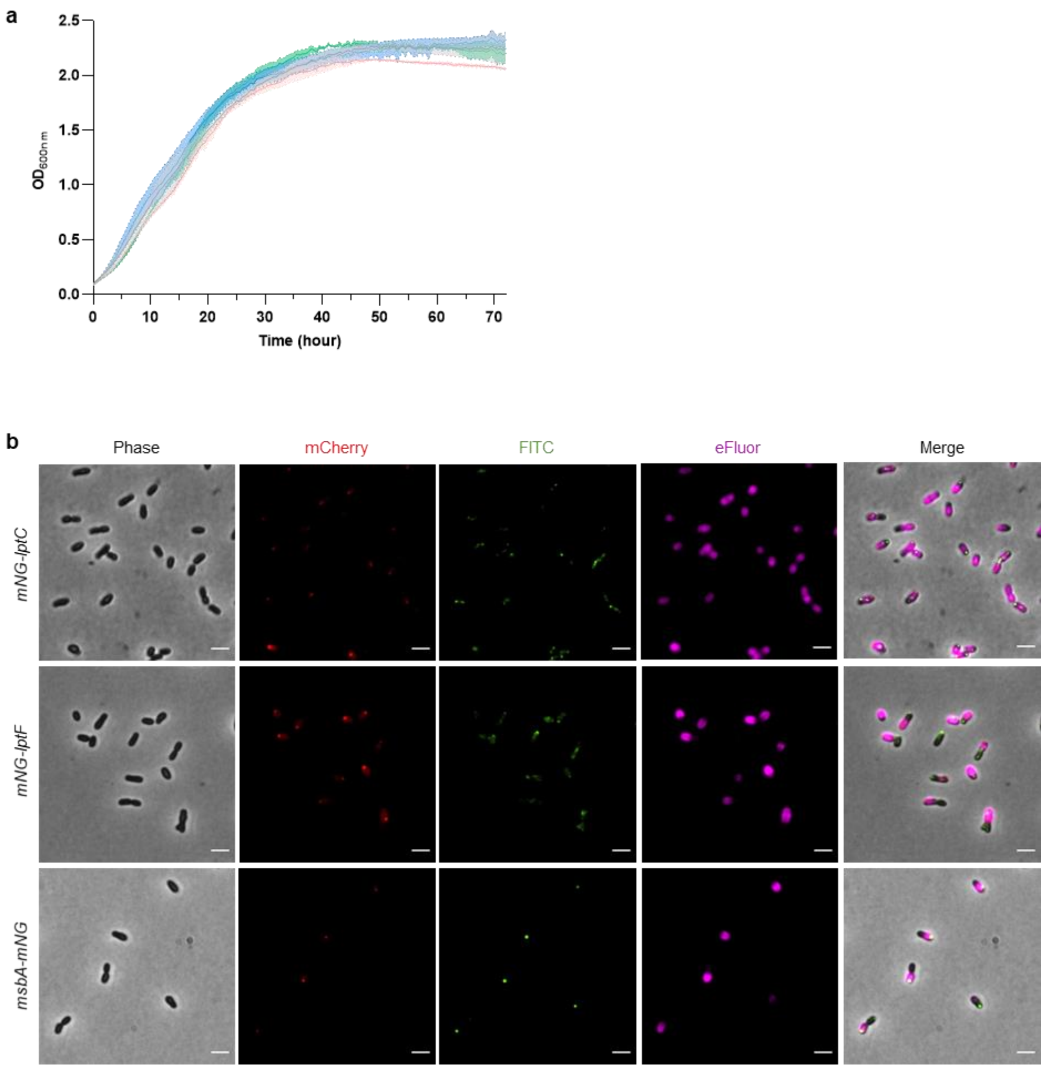

Figure S3 | Growth behaviour of *mNG-lptC*, *mNG-lptF* and *msbA-mNG* strains. a, Exponential phase cultures of WT (grey), *mNG-lptC* (pink), *mNG-lptF* (blue) and *msbA-mNG* (green) were diluted to OD_{600} =0.1 and grown in TSB rich medium at 37°C for 72 h. The OD_{600} was measured every 30 minutes. Dotted lines correspond to one standard deviation and plain line corresponds to the mean of the biological triplicates. N=3. Source data are provided as a Source Data file**. b,** eFluor labelling of *mNG-lptC*, *mNG-lptF* and *msbA-mNG* was performed on early exponential phase culture in strains co-expressing PdhS-mCherry. Bacteria were labelled with eFluor, washed and grown for 2.5 h at 37°C. The eFluor670 (eFluor) covalently binds to the amine groups on the bacterial cell surface, therefore the labelled part corresponds to the old envelope material and the unlabelled part corresponds to newly incorporated material. mNG corresponds to the channel of the fusion with the fluorescent protein mNG. The experiment was performed in biological triplicate for each strain (n=3). Scale bars are 2 µm. Source data are provided as a Source Data file.

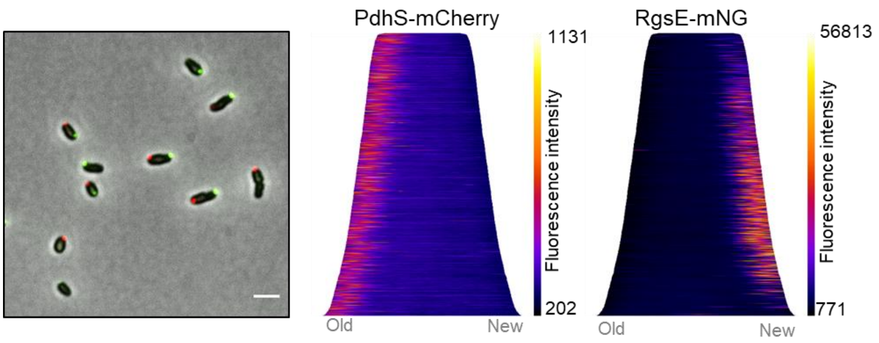

Figure S4 | Polar localization of Rhizobiales growth and septation protein RgsE is conserved in *B. abortus*. Representative merged picture with phase and fluorescent channels of the strain co-expressing PdhS-mCherry (red) and RgsE-mNG (green). The scale bar is 2µm. The right panels show demographic representations of PdhS-mCherry and of RgsE-mNG localization. Cells are aligned based on their length (smallest on top) and pole age using PdhS-mCherry as an old pole marker (old pole on the left). The graphs correspond to one representative experiment (n=1482 bacteria). Fluorescence intensity is represented as a heatmap, the minimum and maximum values represented on the scale were automatically selected to provide the best signal to background by MicrobeJ. Source data are provided as a Source Data file.

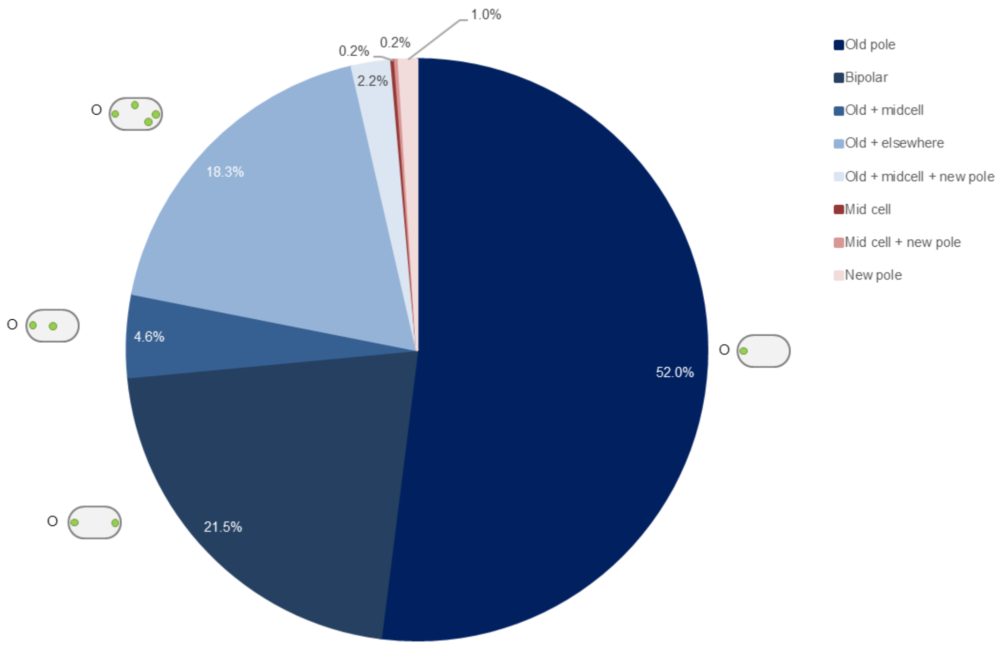

Figure S5 | MsbA localization in *B. abortus*. MsbA is found at the old pole in 98.6% of bacteria. 52% of the cells display only one focus of MsbA at the old pole, 21.5% show a bipolar localization of MsbA, 4.6% display one focus at the mid cell and the old pole, and 18.3% show one focus of MsbA and at least another somewhere else in the bacterium. 2.2% percent of the bacteria display one focus at the old pole, mid cell and at the new pole. Only 0.2% had a focus at the mid cell, 0.2% at the new pole and the division site and 1% had one focus at the new pole only. The analysis was performed by manual counting of bacteria of the strain co-expressing MsbA-mNG and PdhS-mCherry labelled with eFluor. O: Old pole. N=540 bacteria for two biological replicates. Source data are provided as a Source Data file.

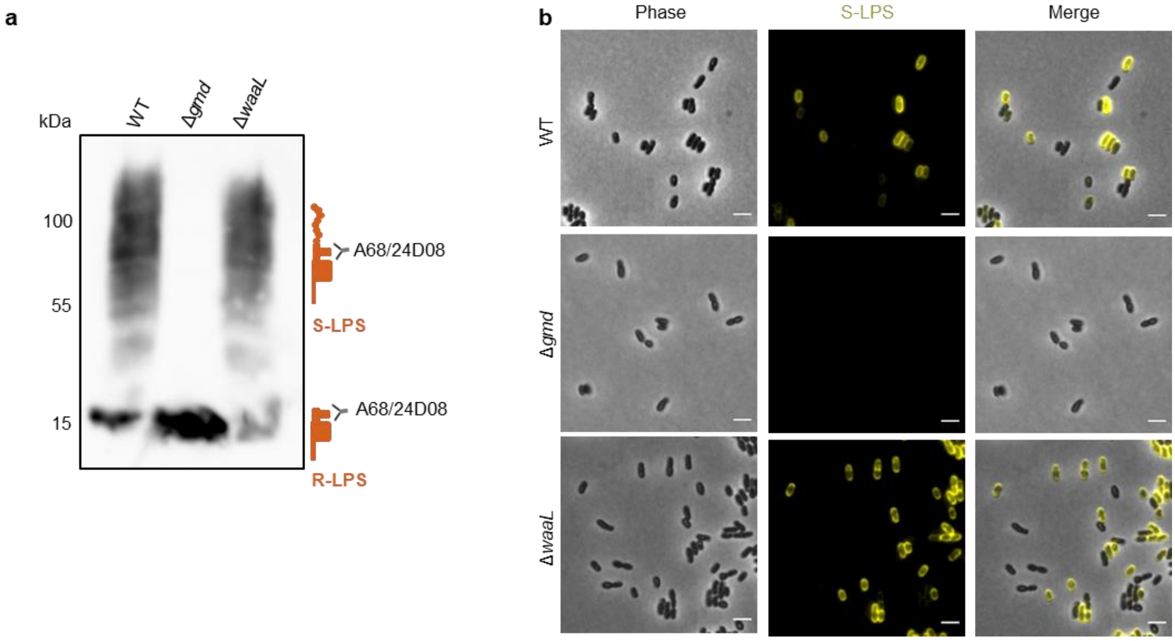

Figure S6 | S-LPS phenotype of *B. abortus* Δ*waaL*. a, Western blot analysis performed using the A68/24D08 mAb targeting the core of both R-LPS and S-LPS (n=3). Source data are provided as a Source Data file. **b**, Immunofluorescence microscopy labelling S-LPS of WT, Δ*gmd* and Δ*waaL* using the mAb A76/12G12. The experiment was performed in biological triplicate (n=3). Scale bars are 2 µm. Source data are provided as a Source Data file.

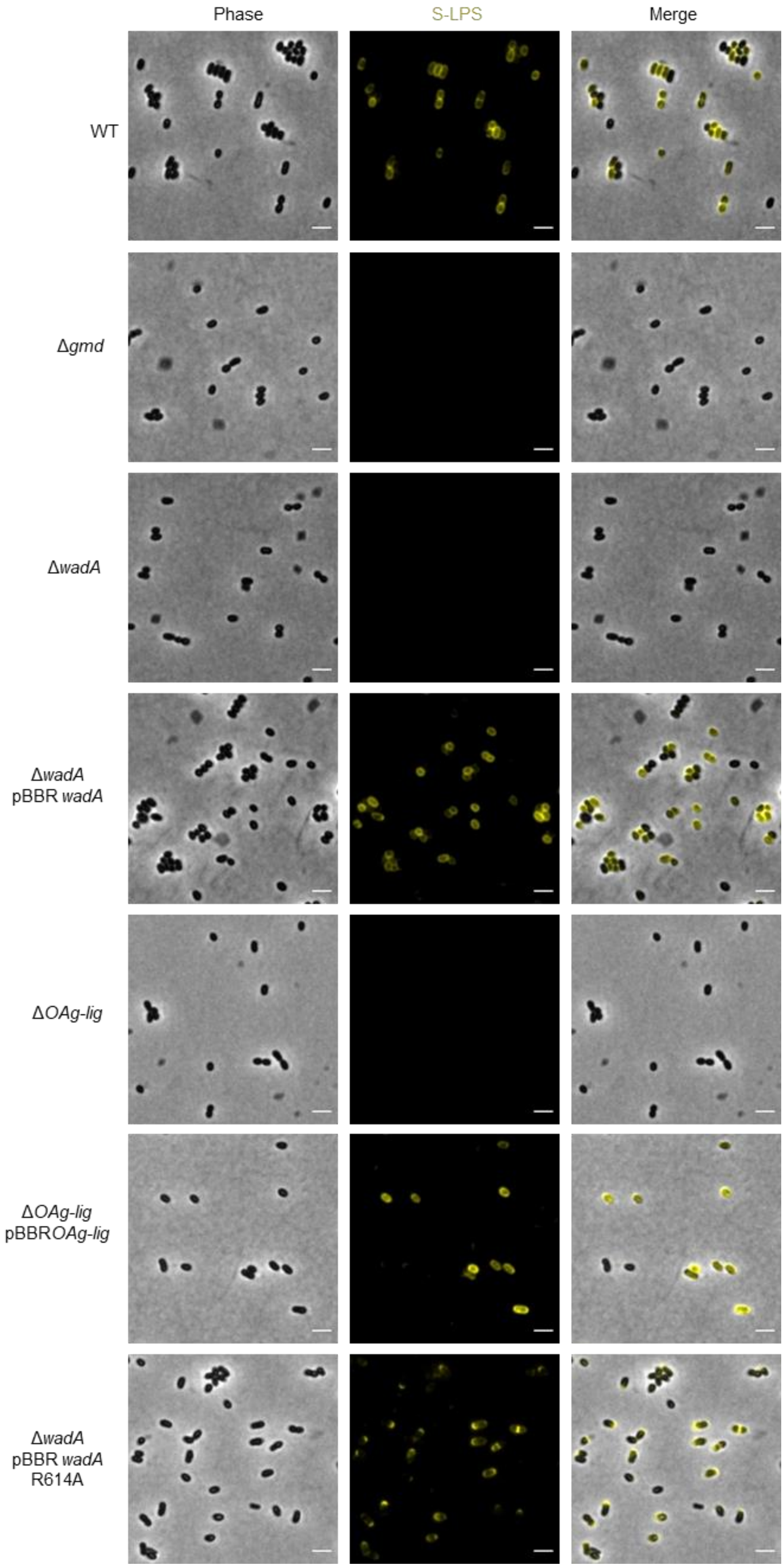

Figure S7 | S-LPS labelling of *B. melitensis* 16M strains. *B. melitensis* strains were labelled with primary mAb targeting the O-antigen (A76/12G12) and a goat anti-mouse antibody coupled to Alexa fluor 514 to detect S-LPS. *Δgmd* was used as a negative control. The experiment was performed in three biological replicates (n=3). Scale bar is 2µm. Source data are provided as a Source Data file.

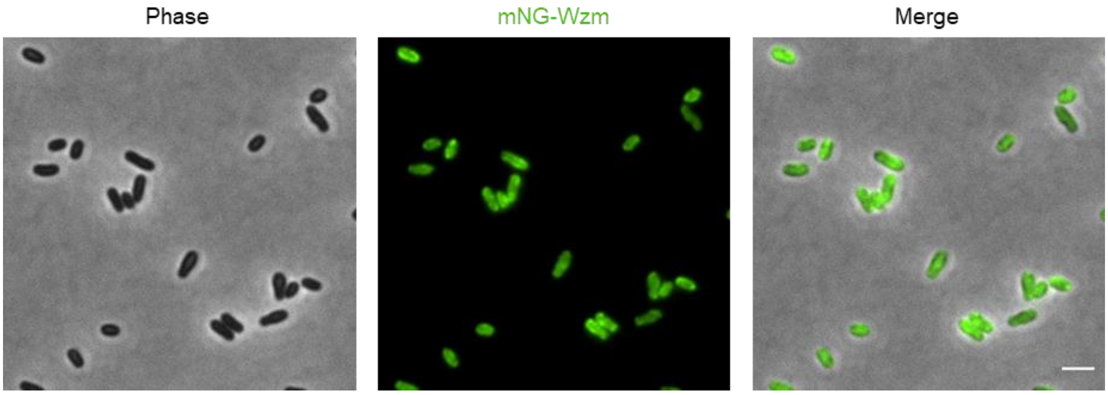

Figure S8 | Wzm appears dispersed surrounding the bacterial cell. Representative image for *mNG-wzm* observed in exponential phase of growth for one biological replicate (n=3). Scale bar is 2 µm. Source data are provided as a Source Data file.

References

1 Vassen, V. *et al.* Localized incorporation of outer membrane components in the pathogen *Brucella abortus*. *EMBO J* **38** (2019). https://doi.org:10.15252/embj.2018100323

2 Haine, V. *et al.* Systematic targeted mutagenesis of Brucella melitensis 16M reveals a major role for GntR regulators in the control of virulence. *Infect Immun* **73**, 5578-5586 (2005). https://doi.org:10.1128/IAI.73.9.5578-5586.2005

3 Kovach, M. E., Phillips, R. W., Elzer, P. H., Roop, R. M., 2nd & Peterson, K. M. pBBR1MCS: a broad-host-range cloning vector. *Biotechniques* **16**, 800-802 (1994).

4 Van der Henst, C. *et al.* Overproduced Brucella abortus PdhS-mCherry forms soluble aggregates in Escherichia coli, partially associating with mobile foci of IbpA-YFP. *BMC Microbiol* **10**, 248 (2010). https://doi.org:10.1186/1471-2180-10-248

5 Deghelt, M. *et al.* G1-arrested newborn cells are the predominant infectious form of the pathogen *Brucella abortus*. *Nat Commun* **5**, 4366 (2014). https://doi.org:10.1038/ncomms5366

6 Cloeckaert, A., Zygmunt, M. S., Nicolle, J. C., Dubray, G. & Limet, J. N. O-chain expression in the rough Brucella melitensis strain B115: induction of O-polysaccharide-specific monoclonal antibodies and intracellular localization demonstrated by immunoelectron microscopy. *J Gen Microbiol* **138**, 1211-1219 (1992). https://doi.org:10.1099/00221287-138-6-1211

7 Cloeckaert, A. *et al.* Protection conferred on mice by monoclonal antibodies directed against outer-membrane-protein antigens of Brucella. *J Med Microbiol* **34**, 175-180 (1991). https://doi.org:10.1099/00222615-34-3-175

8 Bowden, R. A., Cloeckaert, A., Zygmunt, M. S., Bernard, S. & Dubray, G. Surface exposure of outer membrane protein and lipopolysaccharide epitopes in *Brucella* species studied by enzyme-linked immunosorbent assay and flow cytometry. *Infect Immun* **63**, 3945-3952 (1995). https://doi.org:10.1128/iai.63.10.3945-3952.1995

9 Cloeckaert, A., De Wergifosse, P., Dubray, G. & Limet, J. N. Identification of Seven Surface-Exposed *Brucella* Outer Membrane Proteins by Use of Monoclonal Antibodies: Immunogold Labeling for Electron Microscopy and Enzyme-Linked Immunosorbent Assay. *Infection and Immunity* **58**, 3980-3987 (1990).

10 Altschul, S. F. *et al.* Gapped BLAST and PSI-BLAST: a new generation of protein database search programs. *Nucleic Acids Res* **25**, 3389-3402 (1997). https://doi.org:10.1093/nar/25.17.3389

11 Sievers, F. *et al.* Fast, scalable generation of high-quality protein multiple sequence alignments using Clustal Omega. *Molecular Systems Biology* **7** (2011).
