# Supplementary material for: Lipopolysaccharide biosynthesis and traffic in the envelope of the pathogen Brucella abortus
Source: Nat Commun. 2023 Feb 17;14:911. doi: 10.1038/s41467-023-36442-y (PMC9938171; doi:10.1038/s41467-023-36442-y)
Supplement: Supplementary file 2 — Reporting Summary [file 41467_2023_36442_MOESM2_ESM.pdf]

nature portfolio

Corresponding author(s): Xavier De Bolle

Last updated by author(s): Jan 12, 2023

Reporting Summary

Nature Portfolio wishes to improve the reproducibility of the work that we publish. This form provides structure for consistency and transparency in reporting. For further information on Nature Portfolio policies, see our Editorial Policies and the Editorial Policy Checklist.

Statistics

For all statistical analyses, confirm that the following items are present in the figure legend, table legend, main text, or Methods section.

n/a	Confirmed	
☐	☒	The exact sample size (*n*) for each experimental group/condition, given as a discrete number and unit of measurement
☐	☒	A statement on whether measurements were taken from distinct samples or whether the same sample was measured repeatedly
☐	☒	The statistical test(s) used AND whether they are one- or two-sided<br>*Only common tests should be described solely by name; describe more complex techniques in the Methods section.*
☒	☐	A description of all covariates tested
☒	☐	A description of any assumptions or corrections, such as tests of normality and adjustment for multiple comparisons
☐	☒	A full description of the statistical parameters including central tendency (e.g. means) or other basic estimates (e.g. regression coefficient) AND variation (e.g. standard deviation) or associated estimates of uncertainty (e.g. confidence intervals)
☐	☒	For null hypothesis testing, the test statistic (e.g. *F*, *t*, *r*) with confidence intervals, effect sizes, degrees of freedom and *P* value noted<br>*Give P values as exact values whenever suitable.*
☒	☐	For Bayesian analysis, information on the choice of priors and Markov chain Monte Carlo settings
☒	☐	For hierarchical and complex designs, identification of the appropriate level for tests and full reporting of outcomes
☒	☐	Estimates of effect sizes (e.g. Cohen's *d*, Pearson's *r*), indicating how they were calculated

Our web collection on statistics for biologists contains articles on many of the points above.

Software and code

Policy information about availability of computer code

Data collection	No open source or custom code was used to collect data in this study.
Data analysis	The following softwares were used for data analyses: Delta Blast, Blast-P and Clustal Omega (1.2.4) were used for sequences alignments, MicrobeJ (5.13l), a plugin of ImageJ and ImageJ 2.0.0 (Java 1.8.0_0172) were used to analyze microscopy pictures. GraphPad Prism 8 (GraphPad Software) was used to analyse the growth curves. AlphaFold2 (https://colab.research.google.com/github/sokrypton/ColabFold/blob/main/AlphaFold2.ipynb#scrollTo=G4yBrceuFbf3) was used to generate the 3D model of WadA. No custom code was used in this study.

For manuscripts utilizing custom algorithms or software that are central to the research but not yet described in published literature, software must be made available to editors and reviewers. We strongly encourage code deposition in a community repository (e.g. GitHub). See the Nature Portfolio guidelines for submitting code & software for further information.

Data

Policy information about availability of data

All manuscripts must include a data availability statement. This statement should provide the following information, where applicable:

- Accession codes, unique identifiers, or web links for publicly available datasets
- A description of any restrictions on data availability
- For clinical datasets or third party data, please ensure that the statement adheres to our policy

All the data generated in this study have been deposited in the Figshare database at https://doi.org/10.6084/m9.figshare.c.6383685.v1 and source data are provided with this paper.

Human research participants

Policy information about studies involving human research participants and Sex and Gender in Research.

Reporting on sex and gender	N/A
Population characteristics	N/A
Recruitment	N/A
Ethics oversight	N/A

Note that full information on the approval of the study protocol must also be provided in the manuscript.

Field-specific reporting

Please select the one below that is the best fit for your research. If you are not sure, read the appropriate sections before making your selection.

☒ Life sciences ☐ Behavioural & social sciences ☐ Ecological, evolutionary & environmental sciences

For a reference copy of the document with all sections, see nature.com/documents/nr-reporting-summary-flat.pdf

Life sciences study design

All studies must disclose on these points even when the disclosure is negative.

Sample size	All demograph analyses have been performed in biological triplicates and the sample size ranged from 536 to 1482 bacteria. The number of replicates for the other experiments is stated in the "Replication" section below.
Data exclusions	For the demograph analyses, bacteria forming aggregates or displaying no focus for the polar marker (PdhS or PopZ) were excluded from the analysis as they could not be used for the analysis.
Replication	SEM microscopy pictures for gmd disruptant control (Fig. S2b) was performed in biological duplicate and the counting of MsbA foci (Fig. S5) was performed from biological duplicate with 540 bacteria as final count (Fig. S5). Beside this, all experiments were performed in biological triplicate.
Randomization	This aspect is not relevant to our study.
Blinding	Because of the molecular microbiology nature of the work, blinding was not necessary for this study.

Reporting for specific materials, systems and methods

We require information from authors about some types of materials, experimental systems and methods used in many studies. Here, indicate whether each material, system or method listed is relevant to your study. If you are not sure if a list item applies to your research, read the appropriate section before selecting a response.

Materials & experimental systems

n/a	Involved in the study
☐	☒ Antibodies
☒	☐ Eukaryotic cell lines
☒	☐ Palaeontology and archaeology
☒	☐ Animals and other organisms
☒	☐ Clinical data
☒	☐ Dual use research of concern

Methods

n/a	Involved in the study
☒	☐ ChIP-seq
☒	☐ Flow cytometry
☒	☐ MRI-based neuroimaging

Antibodies

Antibodies used

The following in-house monoclonal antibodies targetting LPS were used: A68/24D08, B66/04F09, and A76/12G12. All these antibodies were used undiluted for IF and diluted 100 times for western blot when applicable. The in-house monoclonal antibody A68/7G11/C10 targeting Omp10 of Brucella was used diluted 1000 times for western blot. The anti-3Flag DYKDDDDK (FG4R, ThermoFisher Scientific, catalog number MA1-91878) and was used diluted 1000 times. In-house polyclonal antibody against LptD of Brucella was used diluted 5000 times for western blots.
The following HRP-coupled secondary antibodies from Dako were used for western blot: anti-rabbit Ig (P0217) and anti-mouse IgG P0260). The following fluorochrome-coupled secondary antibody from Life Technologies was used: anti-mouse IgG (H+L) Alexa Fluor 514 (A-31555).

Validation

The A68/24D08 was assessed and validated by Bowden et al 1995 and was latter confirmed by ELISA by Solen-Llorens et al 2014, B66/04F09 was assessed and validated by ELISA by Cloeckaert et al 1991, A76/12G12 was assessed and validated by ELISA and immunoblotting by Cloeckaert et al 1992. The polyclonal rabbit antibody against the N-terminal part of LptD produced was validated by western blot during this study. The reactivity of the antibody was assessed by western blot on lysates of Brucella abortus strains carrying either a wt version of LptD or the 3-Flag tagged fused version of LptD, size change of the band confirmed the specificity of this polyclonal antibody. This data is available in Fig. 1b.
